# Improved dynamic connection detection power in estimated dynamic functional connectivity considering multivariate dependencies between brain regions

**DOI:** 10.1002/hbm.25124

**Published:** 2020-07-09

**Authors:** Somayeh Maleki Balajoo, Davud Asemani, Ali Khadem, Hamid Soltanian‐Zadeh

**Affiliations:** ^1^ Department of Biomedical Engineering, Faculty of Electrical Engineering K. N. Toosi University of Technology Tehran Iran; ^2^ CIPCE, School of Electrical and Computer Engineering University of Tehran Tehran Iran; ^3^ School of Cognitive Sciences Institute for Research in Fundamental Sciences (IPM) Tehran Iran; ^4^ Radiology Image Analysis Lab Henry Ford Health System Detroit Michigan USA

**Keywords:** resting state functional magnetic resonance imaging, dynamic functional connectivity, multivariate dependencies, dynamic connection detectability, hypothesis testing, surrogate data

## Abstract

To estimate dynamic functional connectivity (dFC), the conventional method of sliding window correlation (SWC) suffers from poor performance of dynamic connection detection. This stems from the equal weighting of observations, suboptimal time scale, nonsparse output, and the fact that it is bivariate. To overcome these limitations, we exploited the kernel‐reweighted logistic regression (KELLER) algorithm, a method that is common in genetic studies, to estimate dFC in resting state functional magnetic resonance imaging (rs‐fMRI) data. KELLER can estimate dFC through estimating both spatial and temporal patterns of functional connectivity between brain regions. This paper compares the performance of the proposed KELLER method with current methods (SWC and tapered‐SWC (T‐SWC) with different window lengths) based on both simulated and real rs‐fMRI data. Estimated dFC networks were assessed for detecting dynamically connected brain region pairs with hypothesis testing. Simulation results revealed that KELLER can detect dynamic connections with a statistical power of 87.35% compared with 70.17% and 58.54% associated with T‐SWC (*p*‐value = .001) and SWC (*p*‐value <.001), respectively. Results of these different methods applied on real rs‐fMRI data were investigated for two aspects: calculating the similarity between identified mean dynamic pattern and identifying dynamic pattern in default mode network (DMN). In 68% of subjects, the results of T‐SWC with window length of 100 s, among different window lengths, demonstrated the highest similarity to those of KELLER. With regards to DMN, KELLER estimated previously reported dynamic connection pairs between dorsal and ventral DMN while SWC‐based method was unable to detect these dynamic connections.

## Graphical abstract

This paper presents kernel‐reweighted logistic regression (KELLER) to estimate dFC in resting state functional Magnetic Resonance Imaging (rs‐fMRI) data. KELLER can estimate dFC through estimating both spatial and temporal patterns of functional connectivity between brain regions. The proposed method is compared with SWC and tapered‐SWC with different window lengths using simulated and real rs‐fMRI data.

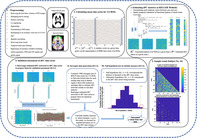

## 1 INTRODUCTION

One of the principal research fields in neuroimaging, particularly resting state functional magnetic resonance imaging (rs‐fMRI), is the analysis of functional connectivity (FC). FC measures the association between intrinsic blood oxygen level‐dependent (BOLD) activities of distant brain regions (B. Biswal, Zerrin, Haughton, & Hyde, 1995; Friston, 2011). Traditionally, researchers have assumed stationarity of FC during scanning period; however, evidences have been reported that inter‐ and intra‐FC of brain networks change over time (Chang & Glover, 2010; Hutchison, Womelsdorf, Gati, Everling, & Menon, 2013; Preti, Bolton, & Van De Ville, 2017). Thus, assessing dynamic pattern of FC has recently become critical for better understanding of the brain function in healthy subjects (Fong et al., 2019; Goldhacker, Tome, Greenlee, & Lang, 2018) as well as its dysfunction in patients with various pathologies such as neurodegenerative diseases (Jones et al., 2012; Wee, Yang, Yap, Shen,, & Alzheimer's Disease Neuroimaging, 2016; Zhu et al., 2019) or neuropsychiatric disorders (Damaraju et al., 2014; Du et al., 2018; Sakoglu et al., 2010; White & Calhoun, 2019).

Dynamic functional connectivity (dFC) has been assessed by different approaches, including time‐frequency coherence analysis (Chang & Glover, 2010), state space model (Kang et al., 2011), time series model (Lindquist, Xu, Nebel, & Caffo, 2014), change point detection methods (Cribben, Haraldsdottir, Atlas, Wager, & Lindquist, 2012; Y. Xu & Lindquist, 2015), regression models with regularization terms (Cai et al., 2018; A. Liu, Chen, McKeown, & Wang, 2015; Monti et al., 2014), and sliding window correlation (SWC) method (Allen et al., 2014; Handwerker, Roopchansingh, Gonzalez‐Castillo, & Bandettini, 2012; Hutchison et al., 2013; Iraji et al., 2019). The SWC approach is a widely applied method in the literature because of its simplicity in both concept and application (Preti et al., 2017).

While dFC studies have recently drawn increasing attention, statistical assessment of the results to capture the underlying dynamic pattern from rs‐fMRI data is of great importance. The spurious fluctuations due to inherent noise in the rs‐fMRI data, low signal‐to‐noise ratio, and physiological artifacts can easily result in false dynamic connections, which are not originated form neural interactions. In addition, how dFC is estimated is influential in the detection of statistically significant dynamic connections (Hindriks et al., 2016; Lindquist et al., 2014; Savva, Mitsis, & Matsopoulos, 2019). For example, Hindriks et al., (2016) have claimed that it is impossible to detect dynamic connections using the SWC method in individual sessions through simulation studies and validated this claim by using both the rs‐fMRI data of the human and the macaque; however, they have reported that averaging the statistical measure across subjects/sessions could increase the power of detecting dynamic connections. In another study, Savva et al., (2019) have shown that mutual information and variation of information yield most consistent results by achieving high reliability with respect to dFC estimations for different window sizes in comparison with correlation metrics such as Pearson correlation, Spearman and Kendall correlation, and Pearson and Spearman partial correlation. Thus, their findings suggested that how dFC is estimated, greatly affects the power of detecting dynamic connections. In consequence, it has recently become critical to determine whether the estimated dFC is in fact due to neuronal interactions or random noise (Hindriks et al., 2016; Kudela, Harezlak, & Lindquist, 2017; Leonardi & Van De Ville, 2015; Zalesky, Fornito, Cocchi, Gollo, & Breakspear, 2014). Therefore, detection of statistically significant dynamic connections is essential for dFC studies.

An appropriate statistical framework is required to determine whether the observed variation in the FC time series can be characterized as dynamic pattern or whether it is due to statistical uncertainty (Hindriks et al., 2016; Sakoglu et al., 2010). To this end, a commonly used approach is to calculate a test measure that characterizes the fluctuation in the FC time series and subsequently applying a statistical hypothesis test. In this framework, the null hypothesis states that the estimated dFC time series is static and is evaluated on the basis of the distribution of the calculated test measure. In the literature, several test measures have been proposed to test the presence of dynamicity in the estimated dFC time series, including the variance of the dFC time series (Hindriks et al., 2016; Sakoglu et al., 2010), a linear measure based on the dFC time series' Fourier transform (Handwerker et al., 2012), and a nonlinear measure (Zalesky et al., 2014). Since the null distributions of the measures cannot be analytically derived, surrogate data, produced based on the statistical properties of the observed data, are used and dynamic connectivity tested based on a test statistics measure that reflects the dynamicity of the estimated dFC time series (Pereda, Quiroga, & Bhattacharya, 2005). Considering the variance of the dFC time series (*σ*^2^) as the test measure, in the absence of dynamicity (null hypothesis), this measure is expected to be only due to statistical uncertainties and remain relatively small over time. On the other hand, in the presence of dynamicity (alternative hypothesis), this measure will not be only due to statistical uncertainties and becomes relatively large. In other words, the variance under the null hypothesis is positive but statistically smaller than that under the alternative hypothesis. Consequently, if the variance of the estimated dFC is located in the upper five percentile of the null distribution, the null hypothesis will be rejected with *p*‐value <.05. This is an evidence for the presence of dynamicity in the estimated dFC time series (Chang & Glover, 2010; Hindriks et al., 2016; Zalesky et al., 2014).

Since one of the main factors that affects the statistical power in the detection of dynamic connections is how dFC is estimated, developing a powerful method to estimate dFC with high accuracy is of critical importance. The SWC‐based methods, as the conventional methods to estimate dFC, suffer from some limitations that can impact the interpretation of the findings (Hindriks et al., 2016; Savva et al., 2019) as follows:

1. *Low detection power*: SWC method uses equal weights across all observations within a window (Lindquist et al., 2014), which in turn leads to variations in the estimation results (Hindriks et al., 2016; Kudela et al., 2017; Lindquist et al., 2014). In consequence, spurious fluctuations caused by noise can easily show up as dynamic changes in the estimated dFC. Hence, the quality of the estimated dFC has an important effect on the power of detecting dynamic connections. Furthermore, the bivariate nature of SWC, which only captures the strength of association between pairs of brain regions, might be another explanation for this limitation. This is because multiple brain regions are engaged in cognitive tasks and resting state conditions (Anzellotti, Caramazza, & Saxe, 2017; Gallagher & Frith, 2003) and using bivariate measures to estimate interactions between these regions may not explore the neural bases of behavior or cognition. Recent exploration of uncertainty in estimation of dFC has reported issues due to stationarity and statistical testing of dFC (Liegeois, Laumann, Snyder, Zhou, & Yeo, 2017). Parametric approaches show greater power in detecting dFC changes. For example, Liegeois et al., (2017) have suggested that Autoregressive models are powerful tools for exploring the dynamical properties of rs‐fMRI. They also explored different frameworks including phase randomization and autoregressive randomization for generating surrogate data for statistical testing of dFC. Their findings showed that bivariate autoregressive randomization approach is prone to false‐positives compared with phase randomization and multivariate autoregressive randomization approaches.
2. *Appropriate window length*: Setting the length of time window is very critical and affects the connectivity results (Shakil, Lee, & Keilholz, 2016). Using a long window risks to miss fast changes in FC evolution over time, whereas a short window will reduce effective sample size and make the estimation procedure unreliable (Hutchison, Womelsdorf, Allen, et al., 2013). However, some efforts have been made to address the algorithmic selection of the window length to explore dFC (Vergara, Abrol, & Calhoun, 2019). Vergara et al., (2019) proposed to use an averaged SWC, which requires a window length smaller than that of SWC. This is important because shorter windows allow for more accurate estimation of transient dynamicity of FC. Including an averaging step in the processing of SWC as proposed in (Vergara et al., 2019) provides a method for eliminating artifact fluctuations due to windowing compared with the common SWC. In this way, the averaged‐SWC identifies dFC fluctuations better than the common SWC.
3. *Sparsity of dFC networks*: The dFC networks resulting from the SWC method are fully dense because of the presence of noise and other nonneuronal sources that contribute to the acquired BOLD signals, whereas numerous studies have reported that brain networks are of parsimonious structure which enable brain to process and transfer information with high efficiency and low redundancy (Bullmore & Sporns, 2009). Thus, estimation of the dFC network structure is a problem (A. Liu et al., 2015; Monti et al., 2014), which is inherently relevant in the estimation of the network's sparsity.

In this work, we adopt the kernel‐reweighted logistic regression (KELLER) from genetic studies (Song, Kolar, & Xing, 2009), which provides us with a comprehensive framework to estimate dFC networks while overcoming limitations of SWC. We change the definitions of the variables in order to match them with those of the rs‐fMRI data. To address the first mentioned limitation, we model dependencies of brain regions by considering multivariate relations between BOLD signals of pairs of brain regions in KELLER. Moreover, kernel‐reweighted feature of KELLER weighs observations unevenly in each window in a way that the adjacent observations have stronger contributions to the estimation process than the distant observations. Thus, we hypothesize that this modeling leads to a more accurate estimation of the brain dynamic interactions. To overcome the second limitation, KELLER utilizes a sophisticated parameter selection technique based on the Akaike information criterion (AIC). Finally, KELLER ensures the sparsity of the estimated dFC networks by applying an ℓ1‐penalized term in the loss function which effectively yields a sparse network.

To utilize KELLER in estimating dFC from rs‐fMRI data, we define a new time‐varying network model based on temporal modeling of rs‐fMRI time series in which we model the multivariate probability density function (pdf) of the rs‐fMRI time series of all brain regions at each time point by using a pairwise Markov Random Field (MRF) model. In this model, dFC between each pair of brain regions indicates the strength of undirected interaction between them. The MRF model has been appealing in the analysis of complex dependence structures (Kaiser, 2007). We reformulate the multivariate pdf of the rs‐fMRI time series of brain regions into a product of conditional pdfs. As a result, the problem of estimating dFC networks is decomposed into one of estimating a series of distinct and static FC networks. Moreover, this step provides an opportunity to estimate multivariate relations between brain regions by estimating functional interaction pattern of a brain region and other regions simultaneously at each time point, using a neighborhood selection procedure (Song et al., 2009; Wainwright, 2006). The resulting functional pattern of each brain region over time is referred to as dynamic neighborhood vector in the rest of the paper. In this way, we can estimate dFC networks by putting together all estimated dynamic neighborhood vectors related to all brain region with a temporal resolution equal to the sampling rate of the BOLD signal. Subsequently, null hypothesis significance testing based on the amplitude‐adjusted phase randomization procedure surrogate data generation (Betzel, Fukushima, He, Zuo, & Sporns, 2016) is employed for detecting dynamic connections (Chen, Rubinov, & Chang, 2017). One possible approach towards obtaining such a statistical assessment is to use a statistic measure that characterizes the changes in the estimated dFC time series (Hindriks et al., 2016; Savva et al., 2019). Then, the estimated statistic measure is tested through its null distribution approximated using surrogate data to verify the presence of dynamic pattern in the estimated dFC time series. Thus, the second hypothesis in this paper is that utilizing KELLER for estimating dFC networks increases dynamic pattern detectability in estimated dFC time series due to modeling the multivariate relations between BOLD signals of brain regions. Moreover, the ability of KELLER to automatically estimate true sparse structure of dFC network at each time point increases the accuracy of the estimated dFC networks.

As mentioned above, SWC has low power of dynamic connection detection because of weighting all observations equally in each window (Lindquist et al., 2014). This limitation of SWC can be overcome by using tapered windows. Thus, we evaluate the performance of KELLER on a series of simulation studies and real rs‐fMRI data in comparison with the SWC‐based methods including SWC and Tapered‐SWC (T‐SWC) (see Section 2.5). Moreover, since the SWC‐based approaches can only obtain time‐varying estimates of the covariance matrices, for fair comparison, we apply the graphical lasso (Friedman, Hastie, & Tibshirani, 2008) subsequently to learn true sparsity structure in the dFC networks. The combined methods are referred to as SWCGL (SWC and graphical lasso) and T‐SWCGL (T‐SWC and graphical lasso) in the rest of the paper.

Another important issue in the SWC method is the effect of window size on the estimated dFC time series. In the literature, a suitable window for dFC is suggested to be between 30 and 100 seconds (s) (Wilson et al., 2015). On the other hand, a rule of thumb has been proposed by Leonardi and Van De Ville for removing spurious fluctuations due to inappropriate window length. They set window length to 1/f_min_ s or larger in SWC, where f_min_ corresponds to the smallest frequency in the spectrum (Leonardi & Van De Ville, 2015; Zalesky & Breakspear, 2015). The spectrum of fMRI BOLD signals has been proposed to start at 0.01 Hz after studying frequencies dominated by neuronal activity and away from physiological noise such as cardiac and respiratory activities (Chen & Glover, 2015). Moreover, Zalesky and Breakspear (Zalesky & Breakspear, 2015) have provided further statistical support for this rule of thumb, but suggested that if fMRI data has a moderate SNR, the window length of 1/f_min_ s may be overly conservative and in this case, dFC can in theory be detected with much shorter window lengths (e.g., 40 s). They have also suggested that statistical testing and appropriate surrogate data is crucial in this respect. Thus, in this work, to minimize the effect of window length on the capability of SWC‐based methods, we use different window lengths from 20 to 140 s in 20 s steps.

The remainder of the paper is organized as follows. In the next section, we will first introduce the KELLER algorithm in detail for computing dFC networks from rs‐fMRI data. Next, in the Materials and Methods Section, we explain simulation studies as well as real rs‐fMRI data. Then, we describe how to estimate dFC time series by utilizing KELLER and detecting dynamic connections. In the Results Section, we present the results of the simulation studies to evaluate the performance of KELLER in comparison with SWC‐based methods. We also present the findings of applying KELLER on the rs‐fMRI data to study the dynamic behavior of the whole brain in healthy subjects.

## 2 KELLER ALGORITHM

In this section, we introduce the KELLER algorithm (Song et al., 2009) for computing dFC networks from rs‐fMRI data. First, a dFC network model based on temporal modeling of rs‐fMRI time series is described. Afterwards, we explain the core of KELLER for estimating dFC networks as the estimation of a dynamic neighborhood vector. Finally, we discuss how parameters of KELLER are set. In the following, matrices, vectors, and scalars are denoted by boldface capital letters, boldface lowercase letters, and lowercase letters, respectively.

### 2.1 Dynamic FC network model based on temporal modeling of rs‐fMRI time series

Let us consider the representative rs‐fMRI time series of *p* regions of interest (ROIs) at a given time point *t* as a random vector denoted by $y^{\left(t\right)}≔\left(y_{1}^{\left(t\right)},\ldots ,y_{p}^{\left(t\right)}\right)$. We suppose that T observations of ***y*** are available denoted by **Y**∈ℝ_T×*p*_. Representative rs‐fMRI time series of each ROI is extracted as the mean rs‐fMRI time series of all voxels in that ROI. Prior biological knowledge of rs‐fMRI data allows us to hypothesize that there may be a meaningful correlation at a given time point *t* between each pair of $y_{m}^{\left(t\right)}\text{and}\;y_{n}^{\left(t\right)},m,n\in 1:p$. Our objective is to estimate dFC matrices {***θ***^(***t***)^, *t* = 1 : *T*} ***≔*** {***θ***^(**1**)^, …, ***θ***^(***T***)^} between all pairs of *y*^(*t*)^ ∈ *ℝ*_*p*_ over time, that is, an FC matrix for every time point of the rs‐fMRI measurement. To consider multivariate interactions between brain regions in estimating dFC matrices, we used the KELLER algorithm originally proposed in the genetic studies framework as a generative model based on a pair‐wise MRF which represents the multivariate pdf of the random vector ***y***^(*t*)^ at a given time point *t*. To translate KELLER from genetic to rs‐fMRI, we need to define a dichotomized variable $d^{\left(t\right)}≔\left(d_{1}^{\left(t\right)},\ldots ,d_{p}^{\left(t\right)}\right)\in {\mathbb{R}}_{p}$ that classifies each observation vector $y^{\left(t\right)}≔\left(y_{1}^{\left(t\right)},\ldots ,y_{p}^{\left(t\right)}\right)\in {\mathbb{R}}_{p}$ at a given time point *t* to “High” ($d_{m}^{\left(t\right)}=1)$ or “Low” ($d_{m}^{\left(t\right)}=-1)$ level of functional activity ($D\left(y^{\left(t\right)}\right):\text{binarized}\;y^{\left(t\right)}\to d^{\left(t\right)}=\left(d_{1}^{\left(t\right)},\ldots ,d_{p}^{\left(t\right)}\right)\in {\mathbb{R}}_{p},d_{m}^{\left(t\right)}ɛ\;\left\{-1,1\right\}).$ To this end, we normalize all the representative rs‐fMRI time series of the *p* regions of interest (ROIs) separately between zero and one; this normalization does not affect the pair‐wise correlation between the ROIs because the temporal variations of the rs‐fMRI time series are preserved. So, the observed random vector denoted by $y^{\left(t\right)}≔\left(y_{1}^{\left(t\right)},\ldots ,y_{p}^{\left(t\right)}\right)\in {\mathbb{R}}_{p}$ at a given time point *t* will be within the interval (0,1) and then can be dichotomized to “High” or “Low” level of functional activity as $d^{\left(t\right)}≔\left(d_{1}^{\left(t\right)},\ldots ,d_{p}^{\left(t\right)}\right)\in {\mathbb{R}}_{p},d_{m}^{\left(t\right)}ɛ\;\left\{-1,1\right\}$ by setting a fixed threshold of 0.5. Note that this thresholding is a kind of adaptive thresholding based on the variation of functional activity in a given ROI, because the representative rs‐fMRI time series of the *p* regions of interest (ROIs) were separately normalized between zero and one before thresholding. Thus, the generative model can be defined based on pair‐wise MRF which represents the joint probability of measured BOLD signal of all ROIs at time point *t*, ***y***^(*t*)^ as follows:

(1) $$P\left(y^{\left(t\right)}|\;\theta^{\left(t\right)}\right)≔\frac{1}{Z\left(\theta^{\left(t\right)}\right)}\exp\left(\sum_{\left(m,n\right)\;\mathrm{are}\;\text{connected}}{\theta_{\mathrm{mn}}^{\left(t\right)}y}_{m}^{\left(t\right)}y_{n}^{\left(t\right)}\right)$$

where $\theta_{\mathrm{mn}}^{\left(t\right)}$ encodes the undirected interaction between the rs‐fMRI signals of each pair of ROIs (*m* and *n*) at time point *t* $\left(\theta_{\mathrm{mn}}^{\left(t\right)}=\theta_{\mathrm{nm}}^{\left(t\right)}\right)$. In other words, dFC between each pair of brain regions is modeled as $\theta_{\mathrm{mn}}^{\left(t\right)}$ which indicates the strength of undirected interaction between them. The partition function *Z*(***θ***^(*t*)^) in the MRF model normalizes Equation (1) to a probability function. In MRF modeling, the dependencies of a set of random variables are represented by an undirected graph. Therefore, dFC matrices {***θ***^(*t*)^} are expected to be undirected, weighted, and symmetric. The presence of a non‐zero entry in the dFC matrix ***θ***^(***t***)^ means that the fluctuations of the rs‐fMRI time series of the corresponding ROIs are functionally related at a given time point *t*. Changes in the value of parameter $\theta_{\mathrm{mn}}^{\left(t\right)}$ over time is considered as the dynamic interaction between ROIs (*m* and *n*). In other words, if the value of $\theta_{\mathrm{mn}}^{\left(t\right)}$ increases (or decreases), it reflects that the interaction between ROIs (*m* and *n*) becomes stronger (or weaker) or connection between them appeared (or disappeared). Accurate estimation of a series of dFC matrices {***θ***^(*t*)^, *t* = 1 : *T*} ***≔*** {***θ***^(1)^, …, ***θ***^(*T*)^} is the main focus of this study.

From the perspective of the brain functional organization, we impose the following two properties on the estimation procedure of the dFC matrices {***θ***^(*t*)^}. Since the topology of FC networks changes smoothly over time (Lin et al., 2017), the first property is the smoothness of variation in dFC pattern over time. In mathematical terms, smooth changes of dFC networks means that the changes in $\left|\theta_{\mathrm{mn}}^{\left(t\right)}-\theta_{\mathrm{mn}}^{\left(t+1\right)}\right|$ are small over time. Note that current limitation in fMRI recording technology allows dFC methods to capture dynamic variation in FC in the time scale of a single fMRI time frame (typically 1–3 s) up to several minutes (Chen et al., 2017). This property indicates that the change in the dFC network structure is small at adjacent time points because temporally neighboring networks are most likely to contain more common connections than temporally distant networks. Thus, it is reasonable to incorporate the information of neighboring time points in the rs‐fMRI time series to estimate dFC network at each time point. The second property is the sparsity of dFC networks (Achard, Salvador, Whitcher, Suckling, & Bullmore, 2006; Bassett & Bullmore, 2006) which makes it reasonable to force dFC networks to be sparse in the estimation process.

The problem of estimating dFC matrices {***θ***^(*t*)^} with the above properties (smooth temporal variations and sparsity of dFC networks) is not practically feasible by maximizing the log‐likelihood of the joint probability function in Equation (1), $P_{\theta^{\left(t\right)}}\left(y^{\left(t\right)}|\;\theta^{\left(t\right)}\right)$, because of the existence of the partition function *Z*(***θ***^(*t*)^) which equals to the sum of a number of exponential terms. To solve this problem, we use a framework that decomposes the problem of estimating dFC network along two axes. The first axis is time, where we estimate the dFC network at each time point, and the second is space, where we identify all ROIs in the brain which are connected to a specific ROI. We refer to these connected ROIs as the neighbors of that specific ROI. We define the neighbors of each ROI at each time point (neighborhood vector of each ROI is defined as $\left\{\theta_{n^{\prime }}^{\left(t\right)}\right\}$ where *n*′ specifies the set of ROIs except {*n*}, i.e., *n*′ ≔ {1, …, *p*} − {*n*}; *j* = 1 : *p*) and recover dFC network by putting together all these vectors. In other words, in the new framework, we decompose the estimation problem to a set of identical optimization tasks by reformulating the problem. An additional benefit of such reformulation is that we can model the level of activity at each ROI (which is a binary variable $d_{n}^{\left(t\right)}ɛ\;\left\{-1,1\right\}$) as a function of interactions between that ROI and its neighboring ROIs. So, the generative model in Equation (1) can be simplified as a set of conditional probabilities of the level of functional activity at each ROI based on the functional activity of the rest of ROIs at time point *t*. These neighborhood vectors reflect multivariate relations between a brain region and the rest of the brain regions at a given time point t. Afterwards, we join the corresponding dynamic neighborhood vectors to recover the overall dFC network at each time point. It is worth mentioning that dynamic neighborhood vector of each ROI at a given time point *t* defines the multivariate functional pattern of a particular brain region with other brain regions.

### 2.2 Estimation of neighborhood vector

In this new framework, we employ neighborhood selection procedure (Song et al., 2009; Wainwright, 2006) to convert estimation of dFC matrices at each time point to estimation of a sequence of neighborhood vectors $\left\{\theta_{n^{\prime }}^{\left(t\right)}\right\}$. In other words, estimating dFC network is equivalent to recovering the structure of interactions of each ROI (*n*) with the rest of ROIs. In fact, if we can correctly estimate the neighborhood vectors, it will lead to exact recovery of dFC networks. Therefore, the joint probability function in Equation (1) is decomposed into the product of conditional probability functions of $y_{n}^{\left(t\right)}$ given $y_{n^{\prime }}^{\left(t\right)}$ denoted by $P\left(y_{n}^{\left(t\right)}|y_{n^{\prime }}^{\left(t\right)},\theta_{n^{\prime }}^{\left(t\right)}\right),$ which represents the conditional probability of the functional activity of ROI *n* at a time point *t*, given the measured BOLD signal of all ROIs except ROI *n* at time point t, ${\;y}_{n^{\prime }}^{\left(t\right)}≔\left(y_{1}^{\left(t\right)},\ldots ,y_{n-1}^{\left(t\right)},y_{n+1}^{\left(t\right)},\ldots ,y_{p}^{\left(t\right)}\right)ɛR^{p-1}$.

Here, we justify how the joint probability function in Equation (1) is decomposed into the product of conditional probability functions. As mentioned in Section (2.1), if two distinct ROIs *m* and *n* are connected at time point *t*, $\theta_{\mathrm{mn}}^{\left(t\right)}\neq 0,$ otherwise, $\theta_{\mathrm{mn}}^{\left(t\right)}=0\;$. Thus, Equation (1) can be simplified as follows:

(2) $$P\left(y^{\left(t\right)}|\;\theta^{\left(t\right)}\right)≔\frac{1}{Z\left(\theta^{\left(t\right)}\right)}\exp\left(\sum_{n=1}^{p}\sum_{\begin{array}{c} m=1,\\ m\neq n \end{array}}^{p}\theta_{\mathrm{mn}}^{\left(t\right)}y_{m}^{\left(t\right)}y_{n}^{\left(t\right)}\right)$$

(3) $$P\left(y^{\left(t\right)}|\;\theta^{\left(t\right)}\right)≔\frac{1}{Z\left(\theta^{\left(t\right)}\right)}\exp\left(\sum_{n=1}^{p}{<\theta }_{n^{\prime }}^{\left(t\right)},y_{n^{\prime }}^{\left(t\right)}>y_{n}^{\left(t\right)}\right)$$

where 〈***v***_**1**_, ***v***_**2**_〉 = ***v***_**1**_^*T*^***v***_**2**_ is the inner product. We use *n*′ to determine all ROIs excluding {n}, that is, *n*′ ≔ {1, …, *p*} − {*n*}; *n* = 1 : *p*. Now, we can rewrite this joint probability function by using only conditional probabilities. Based on the chain rule, it is proven that $P\left(X_{1},\ldots ,X_{N}\right)=\prod_{i=1}^{N}P\left(X_{i}|\overset{i-1}{\underset{j=1}{⋂}}X_{j}\right)$ and the above equation based on the conditional probabilities would be:

(4) $$P\left(y^{\left(t\right)}|\;\theta^{\left(t\right)}\right)≔\prod_{n=1}^{p}P_{\theta^{\left(t\right)}}\left(y_{n}^{\left(t\right)}|\overset{n-1}{\underset{m=1}{⋂}}y_{m}^{\left(t\right)}\right)$$

Because of the upper‐triangular property of the ***θ***^(*t*)^ matrix, each of the conditional probabilities $P_{\theta^{\left(t\right)}}\left(y_{n}^{\left(t\right)}|\overset{n-1}{\underset{m=1}{⋂}}y_{m}^{\left(t\right)}\right),n=1:p$ is the probability of the functional activity of each ROI at a time point *t*, given the measured BOLD signals of the rest of the ROIs at time point t, $y_{n^{\prime }}^{\left(t\right)}$.

As described in Section 2.1, in the new framework adopted from the KELLER algorithm, we used a binary block to define the level of functional activity of each ROI at every time point and assumed a linear relationship between the functional activity level of ROI *n* and those of all other ROIs except ROI *n* at time point *t* and defined $d_{n}^{\left(t\right)}$ as a binary variable of $y_{n}^{\left(t\right)}$ ($D\left(y^{\left(t\right)}\right):\text{binarized}\;y^{\left(t\right)}\to d^{\left(t\right)}\left(d_{1}^{\left(t\right)},\ldots ,d_{p}^{\left(t\right)}\right)\in {\mathbb{R}}_{p},d_{n}^{\left(t\right)}ɛ\;\left\{-1,1\right\},n=1:p)$. Since $d_{n}^{\left(t\right)}$ is a binary variable, it would be mathematically possible to assume that $P_{\theta^{\left(t\right)}}\left(d_{n}^{\left(t\right)}|y_{n^{\prime }}^{\left(t\right)}\right)\;\text{and}\;d_{n}^{\left(t\right)}ɛ\;\left\{-1,1\right\}$ take the form of a logistic regression because their log‐odds ratio is affine, that is, $\log\left(\frac{P\left(x\right)}{1-P\left(x\right)}\right)=\beta_{0}+\beta_{1}x$. Solving this for *P*, it gives $P\left(x|\beta \right)=\frac{1}{1+e^{-\left(\beta_{0}+\beta_{1}.x\right)}}$ (Banerjee, 2007). In our case, the log‐odds ratio follows the following equation:

(5) $$log\left(\frac{p}{1-p}\right)=\log\left(\frac{P_{\theta^{\left(t\right)}}\left(d_{n}^{\left(t\right)}=1|y_{n^{\prime }}^{\left(t\right)}\right)}{P_{\theta^{\left(t\right)}}\left(d_{n}^{\left(t\right)}=-1|y_{n^{\prime }}^{\left(t\right)}\right)}\right)=2{<\theta }_{n^{\prime }}^{\left(t\right)},y_{n^{\prime }}^{\left(t\right)}>$$

where *ℓ* is the log‐odd, and 〈***v***_**1**_, ***v***_**2**_〉 = ***v***_**1**_^*T*^***v***_**2**_ is the inner product. Thus, in our case, all conditional probabilities follow the logistic function as:

(6) $$P_{\theta_{n^{\prime }}^{\left(t\right)}}\left(d_{n}^{\left(t\right)}|y_{n^{\prime }}^{\left(t\right)}\right)=\frac{1}{1+\exp\left(-2d_{n}^{\left(t\right)}〈\theta_{n^{\prime }}^{\left(t\right)},y_{n^{\prime }}^{\left(t\right)}〉\right)}=\frac{\exp\left(2d_{n}^{\left(t\right)}〈\theta_{n^{\prime }}^{\left(t\right)},y_{n^{\prime }}^{\left(t\right)}〉\right)}{\exp\left(2d_{n}^{\left(t\right)}〈\theta_{n^{\prime }}^{\left(t\right)},y_{n^{\prime }}^{\left(t\right)}〉\right)+1}$$

In this model, $\theta_{n^{\prime }}^{\left(t\right)}=\left\{\theta_{\mathrm{mn}}^{\left(t\right)}|m\in n^{\prime },\theta_{\mathrm{nn}}^{\left(t\right)}=0\;\right\}$ as parameters of the model is a *p‐1* dimensional neighborhood vector of the n^*th*^ ROI at time point *t* and $y_{n^{\prime }}^{\left(t\right)}$ is the set of predictors of the model.

Therefore, the estimation of the dFC networks at each time point is decomposed into estimation of *p* dynamic neighborhood vectors. The sequence of dynamic neighborhood vectors $\left\{\theta_{n^{\prime }}^{\left(t\right)}\right\}\;$ are quantified by considering the following negative log‐likelihood function:

(7) $$\gamma \left(\left\{\theta_{n^{\prime }}^{\left(t\right)}\right\};y^{\left(t\right)}\right)=-\log\left(P_{\theta_{n^{\prime }}^{\left(t\right)}}\left(d_{n}^{\left(t\right)}|y_{n^{\prime }}^{\left(t\right)}\right)\right)$$

It is not possible to estimate $\left\{\theta_{n^{\prime }}^{\left(t\right)}\right\}$ by directly minimizing Equation (7) which is the negative log‐likelihood based on only one measurement of variables at each time point. On the other hand, even if we could estimate $\left\{\theta_{n^{\prime }}^{\left(t\right)}\right\}$, Equation (7) would ensure none of the both previously mentioned properties of dFC networks. In order to estimate $\left\{\theta_{n^{\prime }}^{\left(t\right)}\right\}$ using Equation (7) and ensure that the estimated $\left\{\theta_{n^{\prime }}^{\left(t\right)}\right\}$ varies smoothly over time, we introduce the following kernel reweighted function ***w***^(*t*)^(t*):

(8) $$w^{\left(t\right)}\left(t^{*}\right)=k_{h}\left(t^{*}-t\right)/\sum_{t^{*}=1}^{T}k_{h}\left(t^{*}-t\right)$$

where $k_{h}\left(∙\right)=k\left(\frac{∙}{h}\right)$ is a nonnegative symmetric kernel and *h* is a bandwidth parameter that controls the kernel size. In this work, we define ***k***_*h*_(∙) as a radial basis function Gaussian kernel as ***k***_*h*_(*t*) = exp(−*t*^2^/*h*) in a way that the adjacent observations have stronger contributions to the estimation than the distant observations. It is noteworthy that weighting the observations is used in other methods such as short‐time Fourier transform to extract the transient frequency components (Ahmed & Xing, 2009; Song et al., 2009).

(9) $$\gamma \left(\left\{\theta_{n^{\prime }}^{\left(t\right)}\right\};y^{\left(t\right)}\right)=\left(-\sum_{t^{*}=1}^{T}w^{\left(t\right)}\left(t^{*}\right)\log\left(P_{\theta_{n^{\prime }}^{\left(t\right)}}\left(d_{n}^{\left(t^{*}\right)}|y_{n^{\prime }}^{\left(t^{*}\right)}\right)\right)\right)$$

Finally, sparsity is introduced into the model by using an ℓ1‐norm regularization term which assigns a large penalty to vectors with large absolute values. In this way, the penalty term shrinks elements to zero effectively. KELLER minimizes the following loss function:

(10) $${\hat{\theta }}_{n^{\prime }}^{\left(t\right)}={\text{argmin}}_{\theta_{n^{\prime }}^{\left(t\right)}\in R^{p-1}}\left(-\sum_{t^{*}=1}^{T}w^{\left(t\right)}\left(t^{*}\right)\gamma (\theta_{n^{\prime }}^{\left(t\right)};y^{\left(t^{*}\right)})\right)+\delta {\left\|\theta_{n^{\prime }}^{\left(t\right)}\right\|}_{1}$$

We use ‖.‖_1_ for the ℓ1‐norm, ${\left\|v\right\|}_{1}=\sum_{n=1}^{p}\left|v_{n}\right|$. In Equation (10), *δ* ≥ 0 is a constraint to control the magnitude of the estimated dynamic neighborhood vectors and the sparsity of the dFC network defined by combining these neighborhood vectors ${\hat{\theta }}^{\left(t\right)}=\left[{\hat{\theta }}_{1^{\prime }}^{\left(t\right)},\ldots ,{\hat{\theta }}_{n^{\prime }}^{\left(t\right)},\ldots ,{\hat{\theta }}_{p^{\prime }}^{\left(t\right)}\right]$. Now, this model allows for the estimation of dFC networks which have the properties of temporal smoothness and sparsity while providing an accurate estimation of dFC networks with identifying multivariate interactions between ROIs. The model parameters *h* and *δ* are set using the available data as will be described in Section 2.4.

### 2.3 Optimization algorithm

Estimating dFC networks using the neighborhood selection procedure (Song et al., 2009; Wainwright, 2006), described in Section 2.2, requires solving a series of optimization problems given in Equation (10). The ℓ1‐regularized logistic regression problem is a convex and nondifferentiable problem due to the presence of the penalty terms (Song et al., 2009). Such a ℓ1‐regularized logistic regression can be solved by among others, least absolute shrinkage and selection operator (LASSO) (Tibshirani, 1996), grafting (Perkins & Theiler, 2003), generalized LASSO (Roth, 2004), generalized iterative scaling (Goodman, 2002), and projected gradient (PG) (Duchi, Shalev‐Shwartz, Singer, & Chandra, 2008).

In the estimation of dFC networks during resting state or a cognitive process, there are tens of subjects, hundreds of ROIs, and hundreds of time points, and hence about a million optimization problems. Therefore, it is crucial to choose an efficient algorithm for solving the minimizing problems defined in Equation (10) to minimize the overall computation cost. Here, we parallelized the optimization procedure across different ROIs and different time points by implementing the projected gradient (PG) method (Duchi et al., 2008) because of its simplicity and efficiency. Since ℓ1‐regularized logistic regression loss function can be reformulated as a constrained minimization problem, we can rewrite Equation (10) as follows:

(11) $${\hat{\theta }}_{n^{\prime }}^{\left(t\right)}={\text{argmin}}_{\theta_{n^{\prime }}^{\left(t\right)}\in R^{p-1}}\left(-\sum_{t^{*}=1}^{T}w^{\left(t\right)}\left(t^{*}\right)\gamma (\theta_{n^{\prime }}^{\left(t\right)};y^{\left(t^{*}\right)})\right)\;s.t.{\left\|\theta_{n^{\prime }}^{\left(t\right)}\right\|}_{1}\leq C_{\delta }\;$$

where *C*_*δ*_ is the upper bound of the first order norm of $\theta_{n^{\prime }}^{\left(t\right)}$ and determines the area (Ω) that contains all the estimated parameters. A one‐to‐one correspondence exists between the penalty parameter *δ* in Equation (10) and *C*_*δ*_ in Equation (11). In the new formulation, the objective function $L\;\left(\theta_{n^{\prime }}^{\left(t\right)}\right)$ is a convex function and its derivative with respect to vector $\theta_{n^{\prime }}^{\left(t\right)}$ is obtained as follows:

(12) $$\nabla^{\left(t\right)}=\partial L\left(\theta_{n^{\prime }}^{\left(t\right)}\right)=-\sum_{t^{*}=1}^{T}w^{\left(t\right)}\left(t^{*}\right)\partial \gamma \left(\theta_{n^{\prime }}^{\left(t\right)}\right)$$

In the PG method, the parameters are updated in line with a negative gradient. Following an update, if the parameter is outside the Ω area, it is projected back into the Ω area. Otherwise, the algorithm goes to the next step. The basic step in this method, which guarantees its performance, is the projection of the parameter into the Ω area:

(13) $$\theta_{n^{\prime }}^{\left(t\right)}\leftarrow \Pi_{\Omega }\left(\theta_{n^{\prime }}^{\left(t\right)}-\eta \nabla^{\left(t\right)}\right)$$

where *Π*_*Ω*_(*a*) = *argmin*_*b*_{‖*a* − *b*‖ | *b* ∈ *Ω*} is the Euclidean projection of vector *a* into the Ω area (Duchi et al., 2008). The implemented version of PG algorithm for the optimization problem in Equation (11) is described in the Supporting Information (S1). It should be noted that the PG method has several internal parameters, such as *α*, *ε*, *and σ*, which are adjusted in accordance with (Bertsekas, 2016).

### 2.4 Parameter selection

The proposed KELLER method requires two input parameters *h* and *δ*, which can be adjusted using the available data. The parameter *h* is the width of the Gaussian kernel. This parameter is the most important factor in controlling the temporal smoothness of the estimated dFC networks. A large kernel size allows for more observations to be used in the estimation of the dFC networks while increases the possibility of losing rapid changes in the dFC network. On the other hand, a small kernel size increases the sensitivity to rapid changes, while, the estimation variance increases due to a drastic drop in the number of observations used for the estimation.

The parameter *δ* controls the sparsity. In particular, a large *δ* will result in a network that has a high degree of sparsity. Therefore, determination of both *h* and *δ* parameters is very important. We employ a sophisticated parameter tuning technique based on AIC. The use of AIC allows us to estimate the in‐sample prediction error for each choice of parameter *h* and *δ* resulting in a clear comparison across different values of each parameter. For a given range of *h* and *δ* values, an extensive grid‐search is performed and for any pair of *h* and *δ*, we define AIC as:

(14) AIChδ=2T∑n∈1:p∑t=1T∑t*=1Twtt*γθ^−ntyt*+2N

where N is the estimated number of degrees of freedom and equals $\sum_{t=1}^{T}\frac{Nz\left({\hat{\theta }}^{\left(t\right)}\right)}{2}$, *Nz*(.) counts the number of non‐zero entries in ${\hat{\theta }}^{\left(t\right)}$, as the estimated dFC matrix at time point *t*. Finally, a pair of parameters that minimizes AIC is chosen to be the optimal values for the parameters *h* and *δ*. In this way, a clear comparison across different values of each parameter is provided (Hastie, Tibshirani, & Friedman, 2009) and their best values are selected.

It is worth mentioning that the Bayesian information criterion (BIC) has been used to tune hyper‐parameters (Song et al., 2009). However, BIC selects the correct model if an infinite amount of data are available (Burnham & Anderson, 2002) or the correct model is among a set of candidates (Olofsen & Dahan, 2015). Since in our application, there is no guarantee that the correct model belongs to a set of candidates, we use AIC.

### 2.5 Comparison to related work

#### 2.5.1 SWC

SWC uses an overlapping partition of the data to estimate the pairwise correlation (Pearson correlation coefficient) between brain regions. For each window, the cross‐correlation matrix is calculated using only the observations within that window. Then, the window slides along the time series and the calculation is repeated. The resulting connectivity time series is smooth since adjacent windows share all data point except those in the double sliding steps. KELLER is capable of estimating dFC network at each time point which means that it uses the maximum overlap between windows. Therefore, in this work, the two following windows in SWCGL and T‐SWCGL also have the maximum overlap and the sliding step is equal to one sample point. Moreover, to minimize the effect of window length on the capability of the SWC‐based methods, we use different window lengths from 20 to 140 s in 20 s steps. Subsequently, we compare the KELLER results with those of the SWC‐based methods with different window lengths. Moreover, since SWC can only obtain time‐varying estimates of the correlation matrices, for fair comparison, we apply the graphical lasso (Friedman et al., 2008) on the SWC results to learn the sparsity structure in the estimated dFC networks (see Section 2.5.3). This combined method is referred to as SWCGL in the rest of the paper.

#### 2.5.2 Tapered sliding window correlation (T‐SWC)

T‐SWC is identical to SWC but it uses weighted Pearson cross‐correlation. As mentioned previously, SWC uses equal weights for all observations in a window (Lindquist et al., 2014), which in turn leads to variations in the estimation results (Hindriks et al., 2016; Kudela et al., 2017; Lindquist et al., 2014). Consequently, spurious fluctuations caused by noise can easily show up as dynamic changes in the estimated dFC. T‐SWC solves this problem by using a discounting function similar to utilizing kernel functions in KELLER. That is, in T‐SWC, the weights are defined at each window by a diminishing function, which exponentially decreases the contribution of more distant time points so that the correlation coefficients weigh recent events more heavily. In this work, we use T‐SWC presented in (Betzel et al., 2016) while we use different window lengths from 20 to 140 s in 20 s steps. Then, we compare the KELLER results with those of the SWC‐based methods with different window lengths. Moreover, for fair comparison, we also apply the graphical lasso (Friedman et al., 2008) subsequently on the results of T‐SWC to learn the sparsity structure in the estimated dFC networks (see Section 2.5.3). This combined method is referred to as T‐SWCGL in the rest of the paper.

#### 2.5.3 How the graphical lasso was applied on the results of SWC and T‐SWC

To apply graphical lasso on the results of SWC and T‐SWC, we first convert the calculated correlation matrix at the t^th^ window to the covariance matrix by:

(15) $${\mathrm{cov}}_{\mathrm{ij}}^{\left(t\right)}={\mathrm{cor}}_{\mathrm{ij}}^{\left(t\right)}\times \sigma_{i}^{\left(t\right)}\times \sigma_{j}^{\left(t\right)}$$

Here, the ij^th^ element of the covariance matrix is related to the corresponding element of the correlation matrix by the above formula where *σ*_*i*_ and *σ*_*j*_ are the standard deviation (*SD)* of the i^th^ and j^th^ variables at the t^th^ window. Then, the corresponding precision (inverse covariance) matrix at the t^th^ window is estimated while considering sparsity in its structure by using sparse inverse covariance estimation with the graphical lasso proposed by Friedman et al., (2008). The point which should be noted is that the sparsity in KELLER is inherited in the algorithm however, for the SWC‐based methods, we do it as a post‐processing step.

## 3 MATERIALS AND METHODS

### 3.1 Simulated data generation and analysis

In this section, we evaluate the performance of KELLER in estimating the dFC networks in comparison with T‐SWCGL and SWCGL. The objective of the simulation studies is to measure the capability of KELLER in retrieving the underlying dFC patterns as well as the power of KELLER in detecting dynamic connections. The evolution of dFC networks over time is generally smooth (Lin et al., 2017), so we tried to replicate it in the simulated datasets. To satisfy this property, dynamicity in the FC networks can be expressed by the emerging (strengthening) of connections or disappearing (weakening) of connections. Thus, dynamic correlation structure between simulated rs‐fMRI datasets over time is expected to vary smoothly without abrupt changes and to behave as a piece‐wise stationary process.

To generate simulated data, we consider well‐known properties of functional brain organization such as high positive temporal autocorrelation of BOLD signals (B. Biswal et al., 1995; Friston, 2011) and self‐organization and scale‐free characteristics of brain networks (Eguiluz, Chialvo, Cecchi, Baliki, & Apkarian, 2005; Lee et al., 2010; X. Liu, Ward, Binder, Li, & Hudetz, 2014). Since, in the literature (Liegeois et al., 2019; Monti et al., 2014; Rogers, Katwal, Morgan, Asplund, & Gore, 2010; Valdes‐Sosa et al., 2005), the first‐order Vector Autoregressive (VAR) processes are used to evaluate dFC networks, we generated simulated rs‐fMRI data based on the first order VAR process. The VAR process is well suited to the task of producing auto‐correlated multivariate time series as they are capable of encoding autocorrelations within components as well as cross‐correlations across components (Cribben et al., 2012; Monti et al., 2014). In order to evaluate the performance of different methods in estimating dFC networks, simulated rs‐fMRI datasets were generated based on a first order VAR model with pre‐defined temporal autocorrelation structures and modulation (Deler & Nelson, 2001). We studied the performance of the proposed algorithm by using two types of random graphs as the structure of a pre‐defined autocorrelation network: Erdős–Rényi random graphs (Erdos & Renyi, 1959) and scale‐free random graphs obtained by using the preferential attachment model of Barabási and Albert model (Barabási & Albert, 1999). Erdős–Rényi random graphs are the simplest and most widely studied type of random networks while the use of scale‐free networks is motivated by the fact that they resemble some aspects of fMRI networks. For example, previous studies (Eguiluz et al., 2005; van den Heuvel, Stam, Boersma, & Hulshoff Pol, 2008) suggested that the degree distribution of the resting state functional brain organization follows a power law. Moreover, it has been shown that the self‐organization property of the functional brain organization is linked with the power‐law (scale‐free) scaling property of functional brain organization (Gisiger, 2001). In the case of scale‐free networks, the power of preferential attachment (new connection) was set to unity on the rs‐fMRI networks, similar to previous studies (Monti et al., 2014). Additionally, we used pre‐defined temporal modulation of autocorrelation matrices in VAR process to ensure that the dynamicity is inherited in the simulated data with gradual changes within a state and that changes from one state to another state are smooth and thus without any abrupt changes.

**FIGURE 1:**
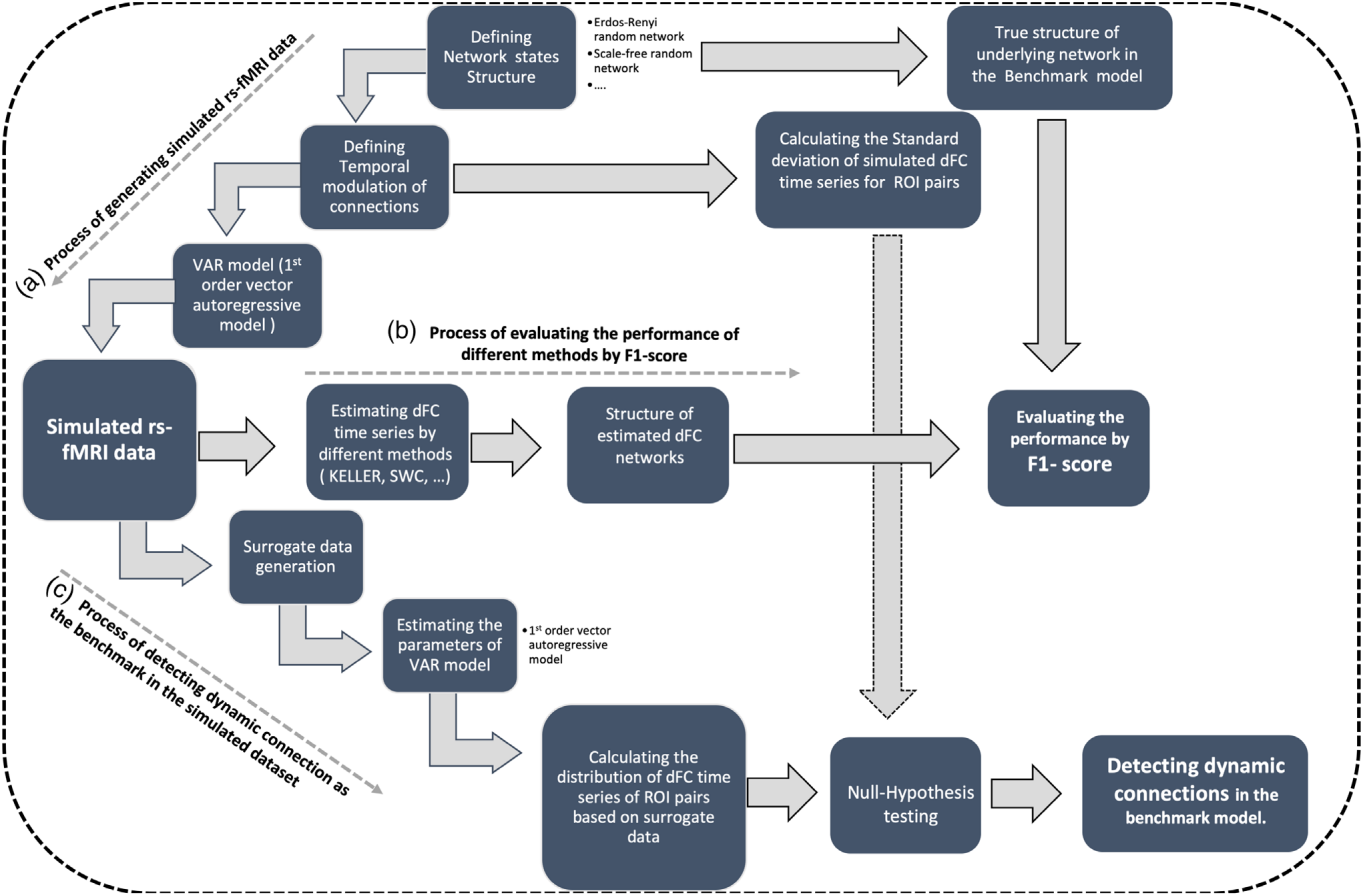
Schematic overview of simulation study including process of generating simulated rs‐fMRI data (a), process of evaluating the performance of different methods by F1‐score (b) and process of detecting dynamic connection as the benchmark in the simulated dataset (c)

Schematic overview of how we generated simulated rs‐fMRI data are illustrated in Figure 1a. We generated simulated dataset based on the first order VAR process while the temporal structure of network and the temporal modulation of connections were predefined. For the simulated data, we considered three states and the correlation structure of each state was randomly generated by Erdős–Rényi random networks (Erdos & Renyi, 1959) or scale‐free random networks (Barabási & Albert, 1999) with 5 nodes in the 0.4–0.7 sparsity range. At each state, the number of simulated observations with repetition time of one second was equal to 100 samples (i.e., total number of observations was 300 and the overall duration was 300 s). Temporal modulation of connections in the VAR process was defined as follows: the strength of the nonzero elements of the network structure (based on random graphs) over time was simulated by a positive slope line for the emerging connections and a negative slope line for the disappearing connections from the range [0.1, 0.75] to avoid abrupt changes. Finally, a vector autoregressive time series for each corresponding connection in the network structure was simulated based on the first order VAR process. Thus, each dataset consisted of 300 samples with 2 change points at times *t* = 100 and 200 s. Moreover, in this simulation study, the dynamicity was simulated within and between states. In fact, gradual changes within a state occur with modeling temporal modulation in the strength of each connection during 100 s of each state with some node‐pair connections emerging or disappearing during each state. This also leads to a change in the structure of connections after 100 s, so that after that time, the brain structure turns smoothly to a new state. The parameters of KELLER were adjusted by AIC as discussed in Section 2.4. In the case of SWCGL and T‐SWCGL, we used various window lengths from 20 to 140 s in 20 s steps, and the penalty parameter in the graphical lasso was estimated by minimizing AIC.

This simulation setup was repeated while the number of nodes increased from 5 to 70 to study the performance of all algorithms. This step is critical as it is often the case that the number of nodes involved in the analysis increases which in turn further increases the difficulty of the estimation procedure. The same parameters of KELLER that were used before were adjusted by AIC as discussed in Section 2.4. But, in the case of SWCGL and T‐SWCGL, we set the window length to 100 s and the penalty parameter in the graphical lasso was estimated by minimizing AIC.

#### 3.1.1 Performance measure to evaluate methods

The goal of this study is to estimate the dFC networks at a sampling rate that leads to a correct estimation of the nonzero elements in the estimated dFC matrices, ${\hat{\theta }}^{\left(t\right)},t=1,\ldots ,T$, so we evaluate the performance of the estimation procedures using an *F1* score (Chinchor, 1992) (Figure 1b). All of the nonzero entries in $\left({\hat{\theta }}_{\mathrm{mn}}^{\left(t\right)}\neq 0\right)$ are assumed to be an edge, and thus, we can define a set that consists of all estimated edges at time point *t*, which is denoted as $E_{\text{edges}}^{\left(t\right)}=\left\{\left(m,n\right),{\hat{\theta }}_{\mathrm{mm}}^{\left(t\right)}\neq 0,m=1:p,n=1:p\right\}$. We define the corresponding set of true edges at time point *t* as $T_{\text{edges}}^{\left(t\right)}=\left\{\left(m,n\right),\theta_{\mathrm{mn}}^{\left(t\right)}\neq 0\right\}$, where *θ*^(*t*)^ denotes the true structure of underlying network state which is completely known in the simulation procedure at time point *t*. Next, we measure the precision, *Pre*^(*t*)^, as the percentage of estimated edges that are present in reality and recall, and *Rec*^(*t*)^as the percentage of true edges estimated by the algorithm. The precision and recall are calculated as follows:

(16) $${\mathrm{Pre}}^{\left(t\right)}=\frac{|E_{\text{edges}}^{\left(t\right)}\cap T_{\text{edges}}^{\left(t\right)}|}{|E_{\text{edges}}^{\left(t\right)}|},{\mathrm{Rec}}^{\left(t\right)}=\frac{|E_{\text{edges}}^{\left(t\right)}\cap T_{\text{edges}}^{\left(t\right)}|}{|T_{\text{edges}}^{\left(t\right)}|}$$

The *F1* score attempts to balance between the *Pre* and *Rec* as a prevalent metric of the performance measure. Finally, the *F*1^(*t*)^ score is defined as

(17) $$F1^{\left(t\right)}=2\frac{{\mathrm{Pre}}^{\left(t\right)}\times {\mathrm{Rec}}^{\left(t\right)}}{{\mathrm{Pre}}^{\left(t\right)}+{\mathrm{Rec}}^{\left(t\right)}}$$

High performance in retrieving the true structure of the network depends on having a high *F*1^(*t*)^ score, which in turn requires that both the precision and recall are also high.

#### 3.1.2 Defining dynamic connections in the benchmark model in the simulation study

Although we simulated dynamicity in the temporal modulation of functional connections as a predefined pattern by gradual emergence or disappearance of connections, we applied a statistical hypothesis analysis on the simulated dFC time series to define connections whose simulated fluctuations in their temporal modulation pattern were due to their dynamic nature. In this analysis, the distribution of the calculated test measure is constructed based on the null hypothesis which corresponds to temporal modulation pattern of the simulated connection being static while the alternative hypothesis corresponds to being dynamic. As illustrated in Figure 1c, we first calculated the variance of the predefined temporal modulation of connections (or equivalently, their *SD*) as the statistical measure (the observed measure). Subsequently, we constructed 500 surrogate sets for the simulated rs‐fMRI data set based on the amplitude‐adjusted phase randomization procedure (Betzel et al., 2016). Next, for each surrogate data, we estimated the parameters of the VAR model which in fact represented how connections in the simulated data were modulated over time. The estimated parameters over time reflected the dynamic pattern of connections in the simulated data. Consequently, we could approximate the null distribution of each parameter of the VAR model by calculating the variance of that parameter over time in 500 surrogate sets. Finally, 95^th^ percentiles of the null distribution were extracted as the significance threshold for rejecting null hypothesis with *p*‐value <.05, that is, the observed measure is greater than 95^th^ percentiles of the distribution. If the null hypothesis is rejected, the simulated temporal modulation pattern for the given connection is dynamic. In this way, we defined the number of dynamic connections in the benchmark model and were able to report the detectability power of dynamic connections by different methods. Then, we estimated dFC time series from the simulated rs‐fMRI data using different methods including KELLER, T‐SWCGL, and SWCGL, and employed statistical hypothesis testing to calculate the percentage of statistically significant dynamic connections. Finally, analysis of variance (ANOVA) in tandem with a post hoc test (permutation test, 100,000 iterations, *p*‐value <.05) was applied to compare the results of KELLER with those of the previous methods.

### 3.2 Real data

We used open access data from the imaging center of the Washington University in St. Louis as one of the 30 international imaging sites involved in the 1,000 Functional Connectomes Project, where for all subjects, T1‐weighted structural as well as rs‐fMRI scans ([dataset] Schlaggar, 2010; B. B. Biswal et al., 2010) were acquired with the same scanning protocol and imaging system. The data available from the Washington University includes 31 healthy subjects (25.1 ± 2.31 years; 14 males). Similar to all international imaging sites involved in the 1,000 Functional Connectomes Project, this center's respective ethics committee approved the submission of the deidentified data obtained with written informed consent from each participant. The rs‐fMRI dataset was acquired using gradient‐echo echo‐planar‐imaging (EPI) pulse sequence and 3 Tesla MRI scanners with eyes open and fixation. During the scanning, 127 volume images were acquired for each subject using the following parameters: repetition time = 2,500 milliseconds, voxel size = 4 × 4 × 4  (mm^3^), field of view = 256 × 256 mm^2^, and 32 slices. Detailed information can be found on the FCP website at http://www.nitrc.org/frs/?group_id=296.

### 3.3 Preprocessing of real data

The rs‐fMRI and anatomical data were preprocessed using Statistical Parametric Mapping (SPM12) and the Data Processing Assistant for Resting‐State fMRI toolbox (DPARSF) (Yan, Wang, Zuo, & Zang, 2016). The preprocessing consisted of the following steps: (a) Removing the first three volumes of each subject's EPI images to remove the BOLD signal transient state; (b) Realigning the remaining EPI volumes to the same subject's mean EPI‐volume using a least square approach with 6° of freedom (rigid body) affine transformation to compensate for the head motion (none of the subjects were excluded due to excessive movement [cumulative translation >2 mm or rotation >2°]); (c) Co‐registering the EPI volumes to the respective structural T1 images; (d) Segmenting T1 images into gray matter (GM), white matter (WM) and cerebrospinal fluid (CSF) using tissue‐probability maps and an affine regularization procedure; (e) Normalizing EPI and T1 images from subject space into Montreal Neurological Institute (MNI) 152 space; (f) Resampling all of the EPI volumes to an isotropic voxel size of 3 × 3 × 3mm^3^; (g) Spatially smoothing the EPI volumes (Gaussian Kernel: FWHM 4 mm); (h)Removing linear temporal trends of the EPI images; (i) Temporally band‐pass filtering the EPI images (0.01–0.1 Hz); and (j) Regressing out the nuisance variables such as the motion parameters (by using Frinston‐24 model), the WM and CSF signals (Kelly, Uddin, Biswal, Castellanos, & Milham, 2008), and the global signal (H. Xu et al., 2018) from the EPI images. A recent study has revealed that global signal regression may greatly influence the estimation of dynamic connection and brain states in the rs‐fMRI studies (H. Xu et al., 2018).

### 3.4 Statistical assessment of estimated dFC time series

To detect true dynamic connections, statistical assessment of the estimated dFC time series is essential for all dFC studies. Therefore, a proper statistical framework should be applied to determine whether the observed variation in the estimated dFC time series can be characterized as dynamic pattern or it is due to statistical uncertainty (Hindriks et al., 2016; Sakoglu et al., 2010). To this end, a commonly used approach is to calculate a test measure that characterizes the fluctuation in the estimated dFC time series by applying a statistical hypothesis test with a null hypothesis which is constructed based on the distribution of the calculated test measure. The null hypothesis states that the estimated dFC time series is static.

#### 3.4.1 Hypothesis testing and statistic measure

In this study, we focus on the variance of the dFC time series (or equivalently, the *SD* of the dFC time series) as a test measure to characterize the fluctuation in the estimated dFC time series. This is the most straightforward and widely used measure in rs‐fMRI studies. In order to obtain dFC time series, we converted the estimated dFC matrix at each time point *t*, ${\hat{\theta }}^{\left(t\right)}$ to a single vector, $V_{\theta }^{\left(t\right)}={\left[{{\hat{\theta }}_{1}}^{\left(t\right)},\ldots ,{{\hat{\theta }}_{n}}^{\left(t\right)},\ldots ,{{\hat{\theta }}_{N}}^{\left(t\right)}\right]}^{T}$ of size *N* × 1 (*N* = *p*(*p* − 1)/2, N = number of ROI pairs) and put these vectors in a matrix, $D=\left[V_{\theta }^{\left(1\right)},\ldots ,V_{\theta }^{\left(t\right)},\ldots ,V_{\theta }^{\left(T\right)}\right],t=1,\ldots ,T$. Thus, D contained the estimated dFC time series of all ROI pairs. Now, the test measure is represented by the *SD* of the estimated dFC time series for the n^th^ ROI pair as:

(18) $$\sigma =\sqrt{\frac{1}{T-1}\sum_{t=1}^{T}{\left({{\hat{\theta }}_{n}}^{\left(t\right)}-\hat{\mu }\right)}^{2}}$$

where ${\hat{\theta }}_{n}=\left[{{\hat{\theta }}_{n}}^{\left(1\right)},\ldots {{\hat{\theta }}_{n}}^{\left(i\right)},\ldots ,{{\hat{\theta }}_{n}}^{\left(T\right)}\right],n=1:N$, is the estimated dFC time series of the n^th^ ROI pair, and $\hat{\mu }$ denotes the sample mean of ${\hat{\theta }}_{n}$. In this model, the null hypothesis (static FC) is defined as: “The *SD* of the dFC time series (*σ*) is only due to statistical uncertainties.” The alternative hypothesis (dynamic FC) is defined as: “The SD of the dFC time series is not only due to statistical uncertainties”. In other words, the *SD* under the null hypothesis is positive but statistically smaller than that under the alternative hypothesis. While testing for the true *SD* equals to zero is theoretically possible, we did not do it because the estimated *SD* will be always positive (nonzero) due to the presence of noise and biological variations. This hypothesis testing is a right‐tailed test; if the *SD* falls within the upper five percentile of the null distribution, we reject the null hypothesis (accept the alternative hypothesis) with a *p*‐value <.05 and conclude that the estimated dFC time series for the n^th^ ROI pair is dynamic. To estimate the distribution of *σ* under the null hypothesis, we use randomized data, known as surrogate data, similar to the analysis of nonstationary time series and dFC (Betzel et al., 2016; Chang & Glover, 2010; Prichard & Theiler, 1994; Zalesky et al., 2014).

#### 3.4.2 Surrogate data generation

To approximate the null distribution, we construct 1,000 surrogate sets of BOLD time series for the 112 ROIs of the Harvard‐Oxford atlas for each rs‐fMRI scan in the data set, using an approach similar to that presented in (Betzel et al., 2016). This approach is based on the amplitude‐adjusted phase randomization procedure in which surrogate BOLD time series are generated with randomized phase, but with the same amplitude distribution so as to preserve the static FC pattern of the real data.

### 3.5 Estimating dFC time series and detecting dynamic connections

**FIGURE 2:**
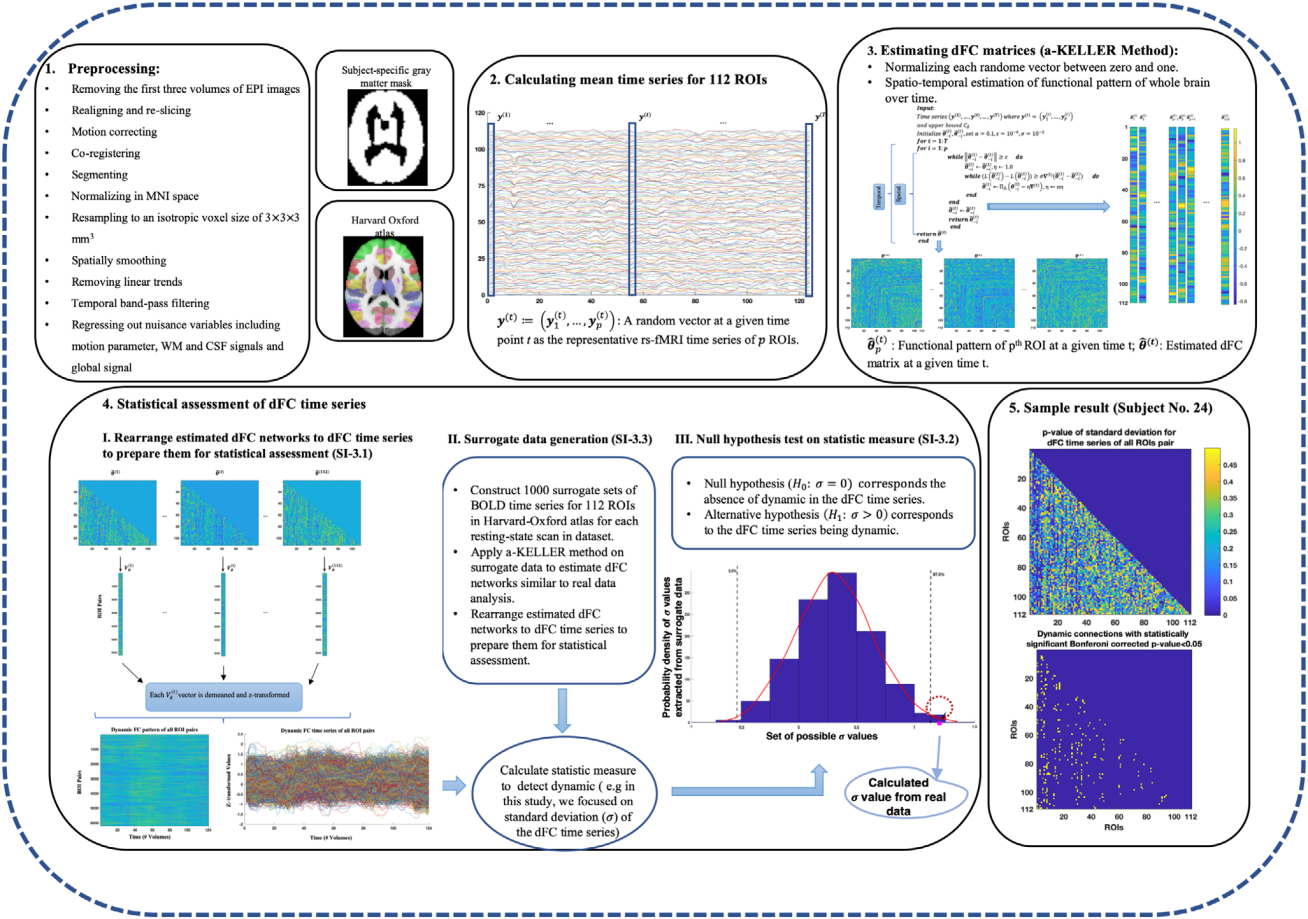
Overview of processing steps on real data to estimate dFC time series using KELLER and to detect dynamic connections. (1) Preprocessing pipeline; (2) Extracting mean time series for 112 ROIs based on Harvard Oxford Atlas; (3) Schematic overview of KELLER to estimate dFC matrices; (4) Statistical assessment of dFC to detect dynamic connections by applying hypothesis testing framework using surrogate data generation approach; (5) Sample result of detected dynamic connection by adjusting the calculated p‐values for multiple comparisons using Bonferroni correction. dFC, dynamic functional connectivity; KELLER, kernel‐reweighted logistic regression

To assess the power of KELLER in detecting dynamic connections and compare with those of SWC based method, we applied KELLER and T‐SWCGL methods on real rs‐fMRI data set. A graphical summery of processing steps on the real rs‐fMRI data based on KELLER method is illustrated in Figure 2. After preprocessing, we estimated individual dFC matrices from 112 extracted time series based on Harvard‐Oxford atlas (Bohland, Bokil, Allen, & Mitra, 2009) by KELLER and T‐SWCGL methods, for each subject. These matrices represent the individual dFC patterns. To reduce dimensional complexity, the upper triangular matrix of the adjacency matrix (size: 112 × 112) excluding the diagonal was vectorized, thereby obtaining a unique vector (size: 6212 × 1) corresponding to each matrix. These adjacency vectors for all time points for each subject were then concatenated to form estimated dFC time series. Then, we analyzed the estimated dFC time series for all 6,216 connection pairs between whole brain regions to detect dynamic pattern from the observed fluctuations of dFC time series. To this end, we calculated the *SD* of the dFC time series as a test measure for each of the 6,216 ROI pairs in 31 subjects. In the case of T‐SWCGL, we obtained dFC time series for each ROI pair using different window lengths from 20 to 140 s in 20 s steps and a step size of one sample (2.5 s). The test measure for each ROI pair in each window length was subsequently calculated. In the following step, we generated, for each subject, 1,000 phase randomized surrogate time series for each ROI in a way that the stationary correlation between every ROI pair was preserved within every set of surrogates. Next, we calculated values of test measure for each of the corresponding 1,000 surrogates with different method including KELLER and T‐SWGL and with different window lengths (including 20 up to 140 s in 20 s steps), for every ROI pair. Finally, for each subject and for each ROI pair, we pooled the values of test measure of all corresponding ROI pairs from 1,000 surrogate data together in order to obtain a *p*‐value for the observed value of test measure in that ROI pair. We also averaged the observed and the surrogate test measure values across subjects and obtained the corresponding *p*‐values by applying hypothesis testing analysis which we refer to as “averaged case” in the following sections. In this way, dynamic connections between all brain region pairs were detected by adjusting the calculated *p*‐values for multiple comparisons using Bonferroni correction.

## 4 RESULTS

### 4.1 Simulation results

**FIGURE 3:**
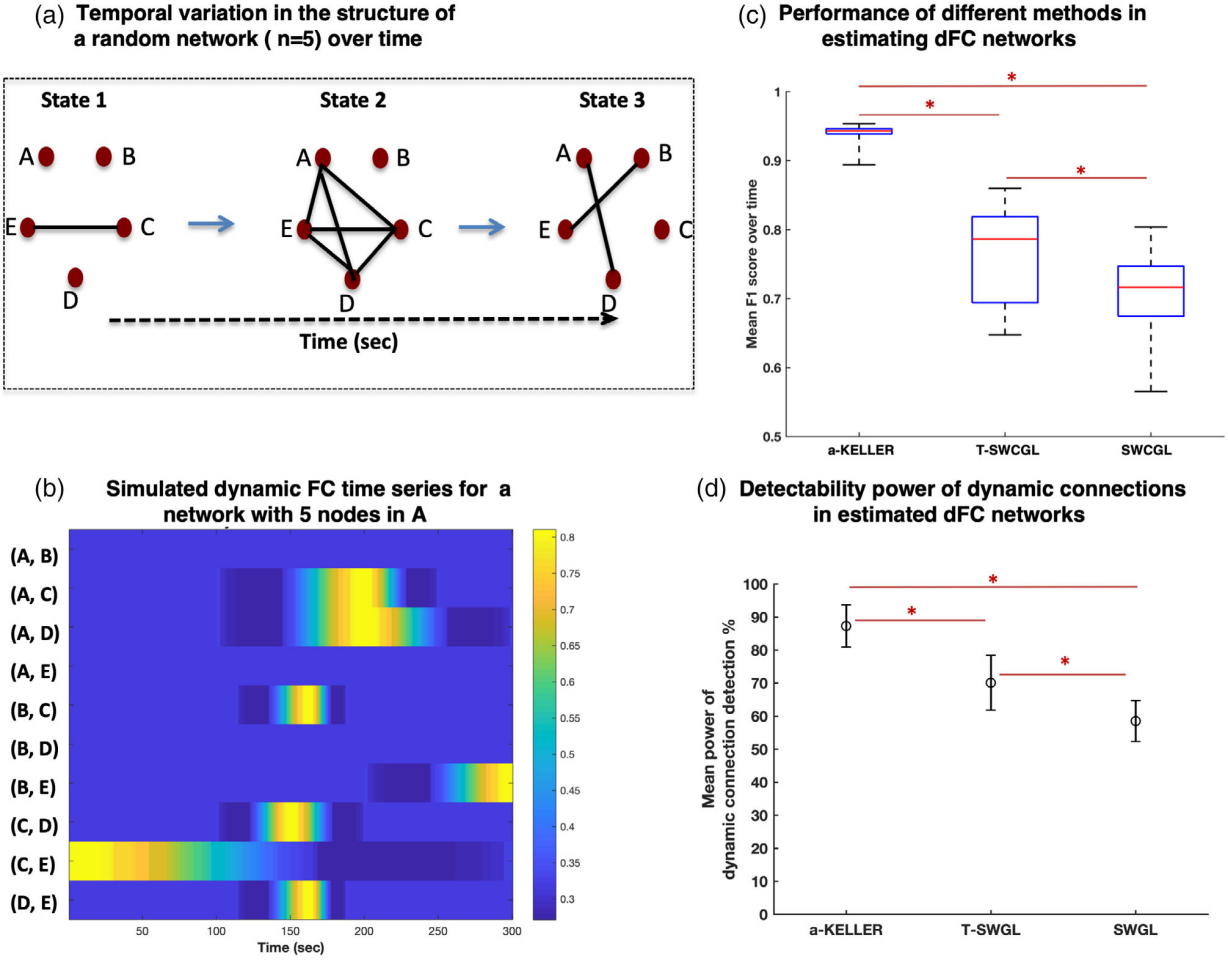
Simulation study with the following setup (network topology = Erdős–Rényi random graph; number of nodes = 5). (a) The sample of simulated dynamic network structure over time, State 1 through State 3, some connections between nodes appeared or disappeared. (b) We vectorized the simulated dFC networks at each time point to construct the evolution of simulated networks over time in terms of the structure and connection's strength in 300 time points for each pair of ROIs. (c) Boxplots of F1 scores as the performance measure of KELLER, T‐SWCGL, and SWCGL in estimating dFC networks. (d) Power of dynamic connections detection calculated by assessing true dynamic fluctuations in the estimated dFC time series by the three methods. In the case of T‐SWCGL and SWCGL, we reported the results for window length of 100 s. *Shows statistical significance. dFC, dynamic functional connectivity; KELLER, kernel‐reweighted logistic regression; T‐SWCGL, tapered sliding window correlation + graphical lasso; SWCGL, sliding window correlation + graphical lasso. Detailed information is provided in the Table 1

A sample of simulated dFC network structure over time using the following setup (network topology = Erdős–Rényi random graph; number of nodes = 5; number of states = 3) as well as simulated dFC time series are presented in Figure 3a,b. The resulting performance of three methods, KELLER, T‐SWCGL, and SWCGL, in estimating dFC networks are demonstrated in Figure 3c based on F_1_ score (mean ± *SD*) over time. In the case of T‐SWCGL and SWCGL methods, we reported the results of setting window length to 100 s. In this plot, the distribution of mean F_1_ score over time was calculated over 100 runs. The results revealed that KELLER (0.94 ± 0.012) estimated the structure of dFC networks over time more accurately than T‐SWCGL (0.76 ± 0.01) with *p*‐value <.001 and SWCGL (0.71 ± 0.02) with *p*‐value <.001 (Table 1).

**TABLE 1:** Performance measure and detectability power of the three different methods in estimating true structure of simulated network

	F1 score (mean ± SD)	*p*‐value	Power of dynamic connection detection (mean ± *SD*)	*p*‐value
KELLER vs.	**0.94 ± 0.012**	<.001	**87.35 ± 6.43**	.001
T‐SWCGL	**0.76 ± 0.01**		**70.17 ± 8.30**	
KELLER vs.	**0.94 ± 0.012**	<.001	**87.35 ± 6.43**	<.001
SWCGL	**0.71 ± 0.02**		**58.54 ± 6.25**	
T‐SWCGL vs.	**0.76 ± 0.01**	.001	**70.17 ± 8.30**	.003
SWCGL	**0.71 *±* 0.02**		**58.54 *±* 6.25**	

*Note:* Simulated network with the following setup: network topology = Erdős–Rényi random graph; number of nodes = 5; Detailed information is given in Figure 3.

Abbreviations: KELLER, kernel‐reweighted logistic regression; *SD*, standard deviation; SWCGL, sliding window graphical lasso; T‐SWCGL, tapered sliding window graphical lasso.

**FIGURE 4:**
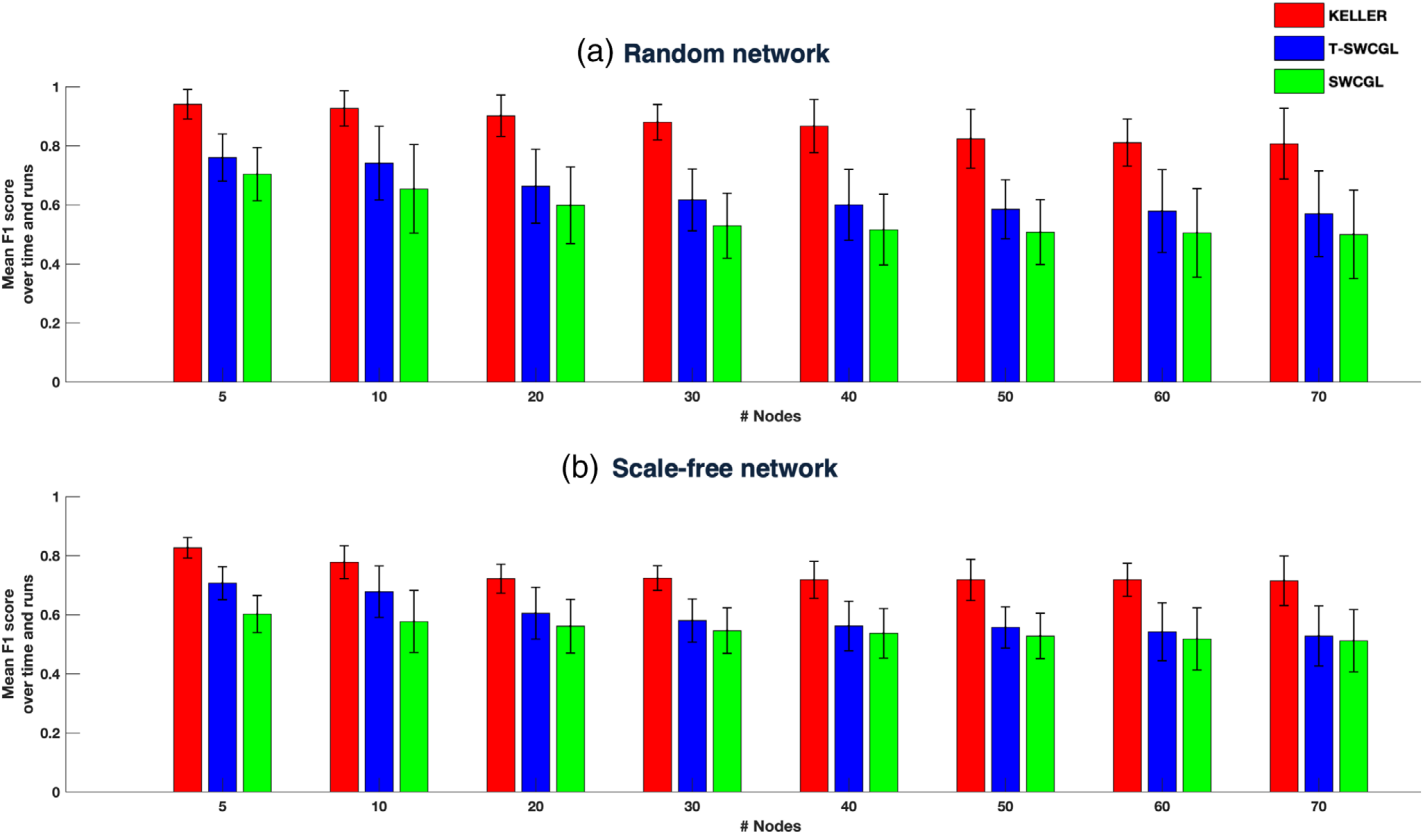
Performance of three different methods in estimating dFC networks in terms of F1 score as the number of nodes increases from 5 to 70 with two different network topology structures: (a) Erdős–Rényi random network; (b) Scale‐free network. The mean F_1_ score over 100 runs and time for KELLER, T‐SWCGL, and SWCGL. In the case of T‐SWCGL and SWCGL, we reported the results for window length of 100 s. Note that there is a drop in the performance of all three methods as the number of nodes increases, but it is less pronounced for the a‐KELLER method. Detailed information is provided in the Supporting Information (Tables S1, S2, and S3). dFC, dynamic functional connectivity; KELLER, kernel‐reweighted logistic regression; T‐SWCGL, tapered sliding window correlation + graphical lasso; SWCGL, sliding window correlation + graphical lasso

**FIGURE 5:**
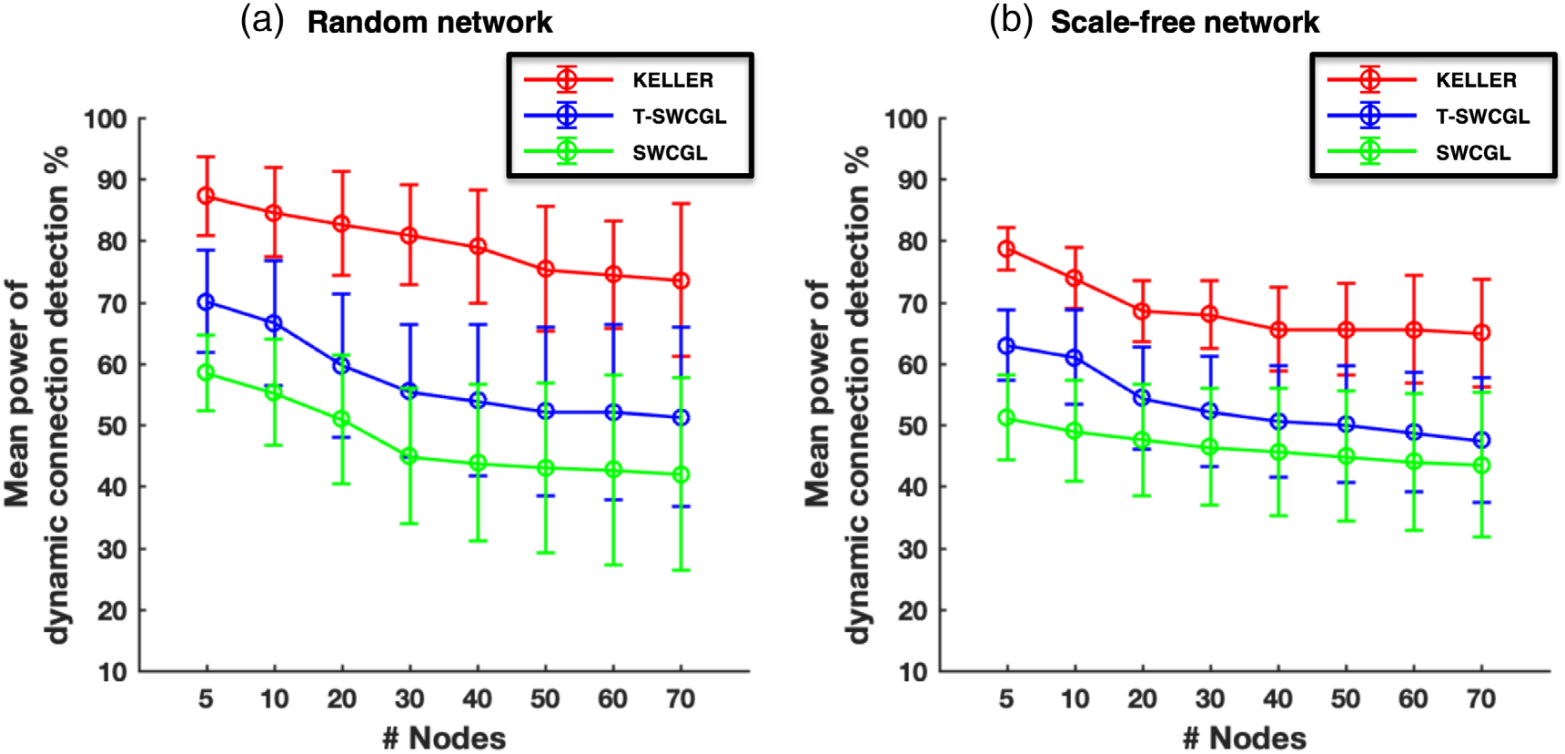
Power of detecting dynamic connections as the number of nodes increases from 5 to 70 with two different network topology structures: (a) Erdős–Rényi random network; (b) Scale‐free network. Mean and *SD* of detectability power over 100 runs and time for estimated dFC time series by KELLER, T‐SWCGL, and SWCGL. In the case of T‐SWCGL and SWCGL, we reported the results for window length of 100 s. Patterns are similar to those in Figure 4. In both network categories, KELLER is more efficient than the other methods in detecting dynamic fluctuations. Detailed information is provided in the Supporting Information (Table S4). dFC, dynamic functional connectivity; KELLER: kernel‐reweighted logistic regression; T‐SWCGL: tapered sliding window correlation + graphical lasso; SWCGL: sliding window correlation + graphical lasso

**FIGURE 6:**
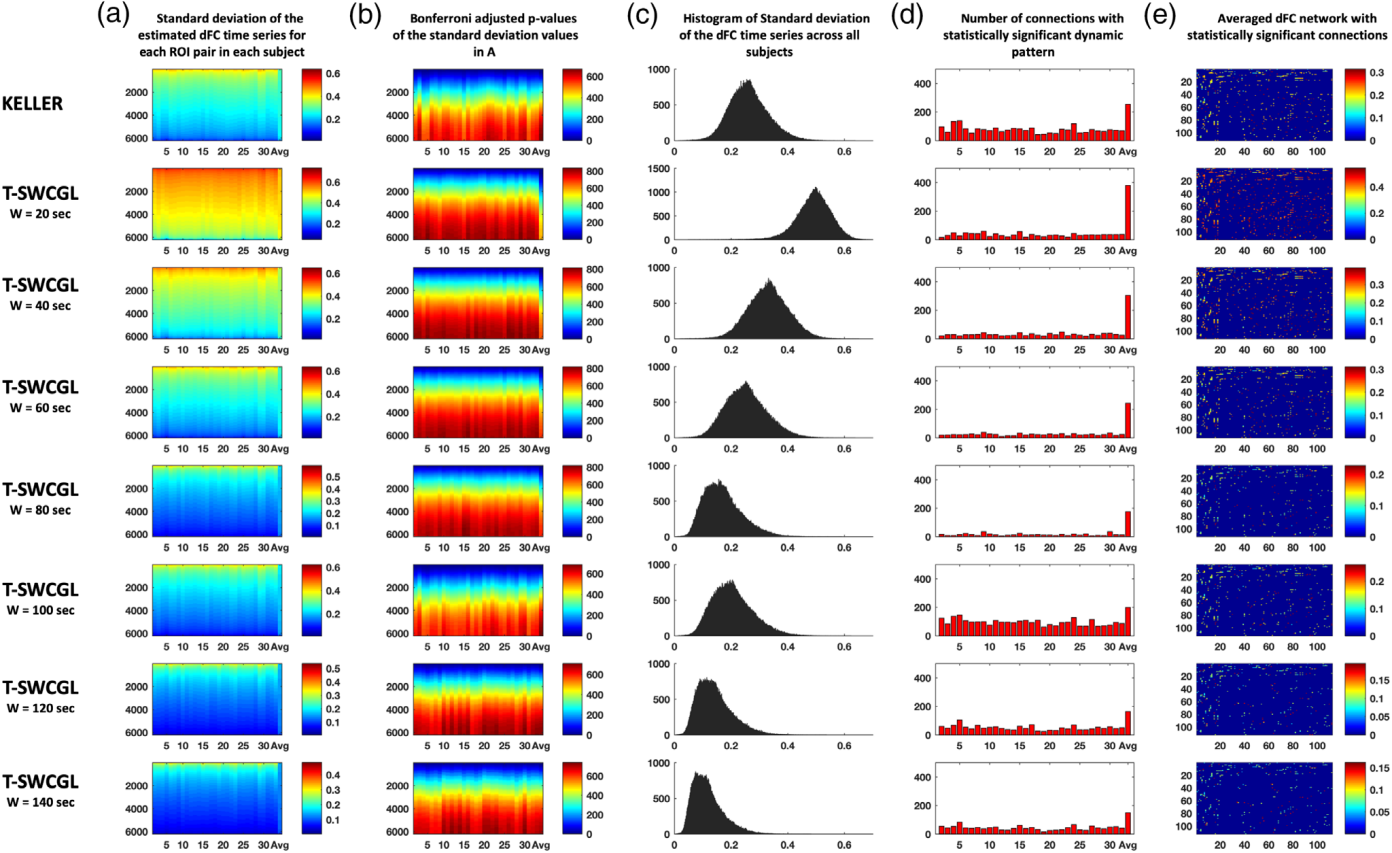
ROI‐pairs analysis of dFC time series by KELLER (first row) and T‐SWCGL with different window lengths including 20 up to 140 s in the following rows. *SD* value of the estimated dFC time series for all ROI pairs for each subject, as well as the averaged case (column a); Corresponding calculated *p*‐values of all those ROI pairs by statistical hypothesis test and adjusted for multiple comparisons using Bonferroni correction, crossed the 5% significance threshold, for each individual subject as well as the averaged case (column b). Histogram of the *SD* value of the estimated dFC time series for all ROI pairs for all subjects (column c). Number of connections with statistically significant dynamic pattern (column d). We found that in the most plots in column d, for an individual, very few connections have significant dynamic pattern while for the averaged case this number increases substantially. However, findings of KELLER and T‐SWCGL with window length of 100 s showed different pattern. In these cases, the number of significant dynamic connection for individuals are more than those of T‐SWCGL with window length of 20, 40, 60, 80, 120, and 140 s. Mean dFC network for the averaged case across subjects with statistically significant dynamic connections adjusted for multiple comparisons using Bonferroni correction (column e). dFC, dynamic functional connectivity; KELLER, kernel‐reweighted logistic regression; T‐SWCGL, tapered sliding window correlation + graphical lasso

**FIGURE 7:**
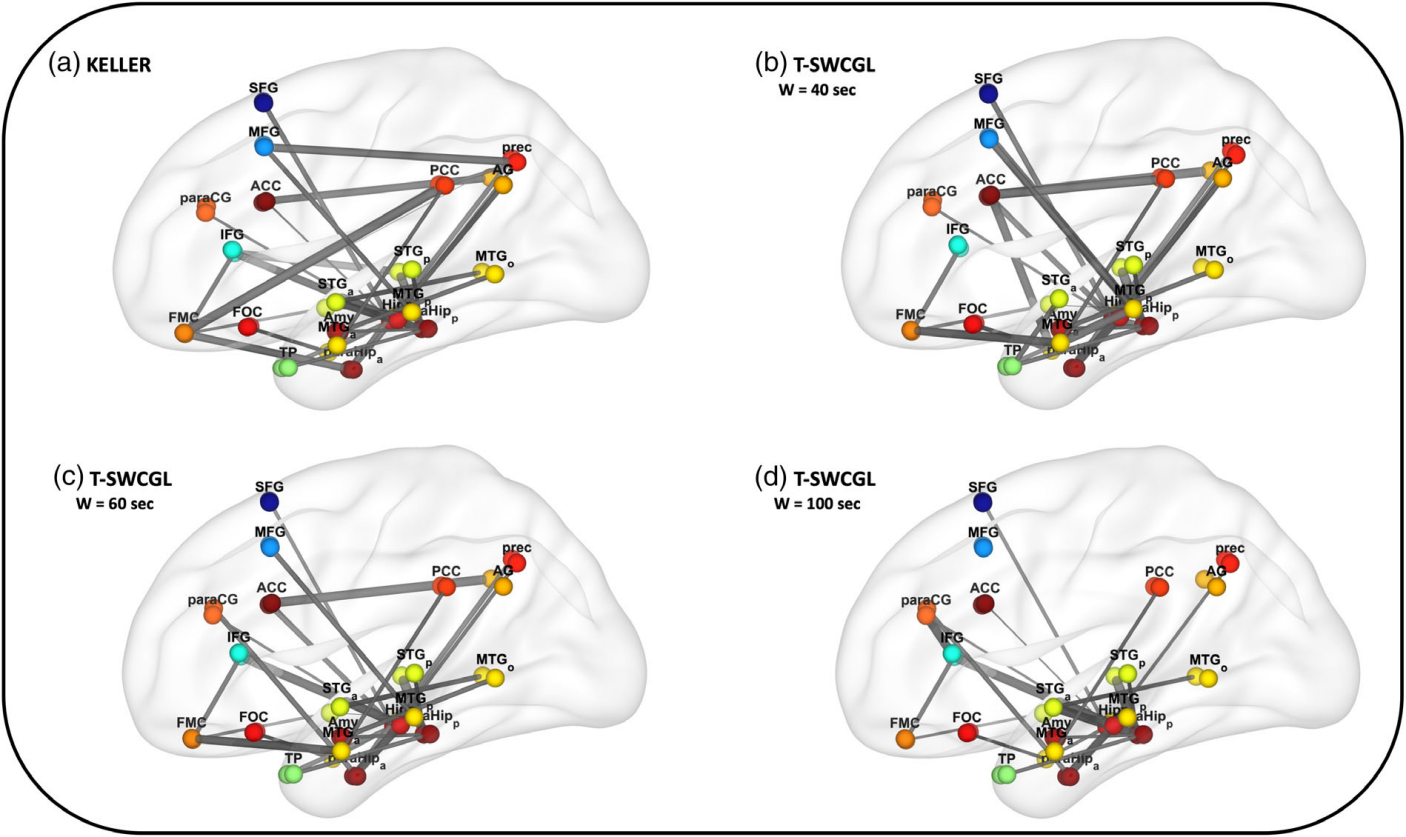
Mean dynamic pattern of default mode networks for the averaged case estimated by (a) KELLER and T‐SWCGL with different window length (w = (b) 40, (c) 60, and (d) 100 s). Note various dynamic connections between anterior and posterior regions of DMN identified by KELLER mostly missed by T‐SWCGL methods. KELLER, kernel‐reweighted logistic regression; T‐SWCGL, tapered sliding window correlation + graphical lasso; DMN, default mode network

In Figure 3d and Table 1, we present the percentage (mean ± *SD*) of statistically significant dynamic connections over 100 runs based on the estimated dFC networks. The results imply that KELLER can detect the dynamic connections by mean statistical power of 87.35% compared with TSWCGL with mean statistical power of 70.17% (*p*‐value = .001) and SWCGL with mean statistical power of 58.54% (*p*‐value <.001). The result from this simulation reveals that KELLER provides more accurate estimates of dFC networks than T‐SWCGL and SWCGL. Additionaly, due to the higher accuracy in the estimation procedure, KELLER has also shown a higher power in detecting dynamic connections than the other methods.

Additionally, we evaluated whether an increase/decrease in window length influenced the results of T‐SWCGL and SWCGL. We ran this simulation study 100 times with 5‐nodes and random network structure. We also applied paired‐samples *t* test to assess if the “average” values obtained by each window length was “significantly” better than those of other window lengths. The results showed that there was no significant difference between the obtained results for different window lengths. However, both T‐SWCGL and SWCGL led to comparable performance with regards to F1‐score and dynamic connection detection power for a window length of 100 sec (Tables 2 and 3).

**TABLE 2:** Performance of SWCGL method in estimating dynamic connections in terms of F1 score, dynamic detection power as the window length was increased from 20 to 140 s with 5‐nodes Erdős–Rényi random network topology structures.

SWCGL	20 s		40 s		60 s		80 s		100 s		120 s		140 s	
Number of windows	281		261		241		221		**201**		181		161	
**F1** (mean ± *SD*)	0.67 ± 0.05		0.68 ± 0.05		0.69 ± 0.04		0.70 ± 0.01		**0.71 ± 0.02**		0.69 ± 0.06		0.65 ± 0.05	
**Dynamic detection** (mean ± *SD*)	54.5 ± 8.2%		55.3 ± 5.5%		56.1 ± 7.9%		57.3 ± 8.1%		**58.5 ± 6.2%**		57 ± 4.9%		53.1 ± 6.9%	
**TRUE**_**sparsit**_	0.6													
**Estimated**_**sparsity**_ (mean ± *SD*)	0.57 ± 0.04		0.54 ± 0.08		0.53 ± 0.06		0.54 ± 0.09		**0.55 ± 0.1**		0.54 ± 0.08		0.54 ± 0.12	
**Confusion matrix**	P	N	P	N	P	N	P	N	P	N	P	N	P	N
**P**	1,931	1,071	1886	1,111	1785	1,035	1,652	900	**1,519**	**766**	1,325	751	1,125	744
**N**	854	3,169	700	2,828	604	2,601	538	2,435	**473**	**2,267**	469	1980	471	1,685

*Note:* The bolded result presents the most significant result.

Abbreviations: *SD*, standard deviation; SWCGL, sliding window graphical lasso; P, positive; N, negative; s, seconds.

**TABLE 3:** Performance of T‐SWCGL method in estimating dynamic connections in terms of F1 score, dynamic detection power as the window length was increased from 20 to 140 s with 5‐nodes Erdős–Rényi random network topology structures.

T‐SWCGL	20 s		40 s		60 s		80 s		100 s		120 s		140 s	
**Number of windows**	281		261		241		221		**201**		181		161	
**F1** (mean ± *SD*)	0.72 ± 0.03		0.73 ± 0.01		0.74 ± 0.02		0.75 ± 0.03		**0.76 ± 0.01**		0.74 ± 0.04		0.7 ± 0.07	
**Dynamic detection** (mean ± *SD*)	66.8 ± 10.3%		67.5 ± 8.9%		67.7 ± 11.3%		68.7 ± 7.5%		**70.1 ± 8.3%**		67.6 ± 6.9%		65.8 ± 12.3%	
**TRUE**_**sparsit**_	0.6													
**Estimated**_**sparsity**_ (mean ± *SD*)	0.58 ± 0.07		0.58 ± 0.08		0.58 ± 0.06		0.59 ± 0.05		**0.59 ± 0.07**		0.58 ± 0.05		0.57 ± 0.08	
**Confusion matrix**	P	N	P	N	P	N	P	N	P	N	P	N	P	N
**P**	2057	894	1923	790	1807	718	1,669	611	**1,548**	**535**	1,356	542	1,158	558
**N**	728	3,346	663	3,149	581	2,919	521	2,724	**444**	**2,498**	438	2,189	438	1871

*Note:* The bolded result presents the most significant result.

Abbreviations: SD: standard deviation; T‐SWCGL, tapered sliding window graphical lasso; P, positive; N, negative; s, seconds.

For a better comparison, we ran the simulations with two different network topology structures while the number of nodes increased from 5 to 70. The results of all algorithms over 100 runs of simulations with two different network topology structures in terms of the mean F_1_ score over time and runs are illustrated in Figure 4. Detailed information including the overall mean and *SD* of *F*_1_^(*t*)^ scores as well as the resulting p‐values for the comparison of the results of different methods are provided in the Table S1. Moreover, the confusion matrices that are the basis of F_1_ score computation are given in the Tables S2 and S3. Note that the performance of all algorithms deteriorates to some extent by increasing the number of nodes and the complexity of network topology structures in the simulations. However, this decrease in the accuracy of estimating dFC networks is less pronounced for KELLER relative to the others. Interestingly, KELLER is the best at keeping its accuracy level as the number of nodes are increased. However, this decline of performance tends to weaken when the number of nodes is larger than 40. This can be explained by the ratio of false positive to false negative values based on the confusion matrix. We provided the related confusion matrix in Tables S2 and S3. It is evident that for the number of nodes larger than 40, this ratio decreases considerably compared with the number of nodes smaller than 40. In other words, if the number of nodes goes to infinity, the ratio of the false positive to false negative values tends to unity. Thus, the performance of methods for the number of nodes larger than 40 can be considered as the steady state performance. As indicated in the Section 2.5.3 the sparsity estimation for the SWC‐based methods was done as a post‐processing step by applying the graphical lasso, and we also compared the estimated sparsity of SWC and T‐SWC with those of KELLER. We reported the mean ± *SD* of the resulting sparsity in the simulated networks by different methods in the simulation study with over 100 runs (Tables S4 and S5). Subsequently, we applied paired–samples *t* test to assess whether there were any significant (*p*‐value <.05) differences between the results. However, no significant differences were found, meaning that the performance of KELLER, with inherited property of forcing the estimated dFC to be sparse, is similar to the performance of SWCGL and the combination of SWC‐based methods with the graphical lasso.

Moreover, for each of the different methods, we assessed the percentage of the observed fluctuations in the estimated dFC time series that were due to its dynamic nature and not statistical uncertainty in comparison with the benchmark model. In Figure 5, we illustrate the mean percentage of statistically significant dynamic connections for each of the different methods, over 100 runs and time based on the estimated dFC networks while the number of nodes increases. Detailed information including the overall mean and *SD* of significant dynamic connections detection power by three methods as well as the resulting *p*‐values for the comparison of the results of different methods are provided in the Table S6. Results in Figure 5 show that their patterns are similar to those obtained in the performance of methods in estimating true structure of dFC networks illustrated in Figure 4. In both simulation studies of random and scale‐free networks, KELLER worked more efficiently in detecting dynamic connections since this method estimated dFC time series more accurately than the other methods as demonstrated in Figure 4.

### 4.2 Experimental results

We applied the KELLER and T‐SWCGL methods to real rs‐fMRI data and compared the results. We did not apply the SWCGL method to real rs‐fMRI data since this method was outperformed in estimating dFC networks over time by the other two methods in the simulation studies.

#### 4.2.1 Detecting dynamic connections in healthy subjects

The first row in Figure 6, depicts the results achieved by KELLER while the following rows show the results of T‐SWCGL with different window lengths from 20 to 140 s, spaced 20 s apart. In Figure 6 (column a), we demonstrated the *SD* of the estimated dFC time series for all ROI pairs of each subject, as well as the averaged case. The related *p*‐values of all those ROI pairs were obtained from statistical hypothesis testing and adjusted for multiple comparisons using Bonferroni correction. These results surpassed the 5% significance threshold for each individual subject as well as in the averaged case are illustrated in Figure 6 (column b). The histogram of the *SD* value of the estimated dFC time series for all ROI pairs for all subjects is depicted in Figure 6 (column c). Figure 6 (column d) shows the number of connections with statistically significant dynamic pattern. We found that in most plots shown in Figure 6 (column d), for an individual, a few connections have significant dynamic pattern while for the averaged case, this number increases remarkably. As previously mentioned, in the averaged case, we averaged the observed and the surrogate test measure values for each ROI pair connection across subjects. We then applied null hypothesis testing to define which connections were dynamic. In fact, the averaging process naturally increased the statistical power of the null hypothesis testing in detecting dynamic connections. However, findings of KELLER and T‐SWCGL with window length of 100 s showed different patterns. The number of significant dynamic connection for individuals in both results obtained by KELLER and T‐SWCGL with window length of 100 s are more than those of T‐SWCGL with window lengths of 20, 40, 60, 80, 120, and 140 s. Figure 6 (column E) demonstrates the mean dFC network for the averaged case across subjects with statistically significant dynamic connections adjusted for multiple comparisons using Bonferroni correction.

To compare the results obtained by KELLER with those of T‐SWCGL considering different window lengths, the similarity between the mean estimated dFC matrix by both methods with statistically significant dynamic connections was calculated for each subject (Table 4). The results revealed that in 68% of the subjects, T‐SWCGL with window length of 100 s had the highest similarity to KELLER. The range of similarity varied from r = 0.37 to r = 0.77 for different window lengths. The findings are in line with the rule of thumb proposed by Leonardi and Van De Ville (2015) which suggests that appropriate SWC window length should be set to 1/f_min_ s or larger, where f_min_ corresponds to the smallest frequency in the spectrum (Leonardi & Van De Ville, 2015; Zalesky & Breakspear, 2015). In this study, f_min_ equals 0.01 Hz and the window length for SWC should be equal to or larger than 100 s. In the averaged case, the highest similarity between KELLER and T‐SWCGL was achieved for a window length of 60 s (r = 0.78), followed by window lengths of 40 and 100 s (r = 0.71).

**TABLE 4:** Similarity between the mean estimated dFC matrix with statistically significant dynamic connections for each subject as well as averaged case by KELLER method and T‐SWCGL with different window lengths.

Window lengths	20 s	40 s	60 s	80 s	100 s	120 s	140 s
**Subject01**	0.09	0.58	0.67	0.51	**0.73**	0.57	0.44
**Subject02**	0.08	0.43	0.49	0.32	**0.57**	0.56	0.56
**Subject03**	0.36	0.45	0.59	0.53	**0.68**	0.49	0.48
**Subject04**	0.21	**0.62**	0.57	0.47	0.57	0.45	0.12
**Subject05**	0.18	0.17	0.55	0.44	**0.62**	0.62	0.59
**Subject06**	0.22	0.26	0.35	0.46	0.55	**0.69**	0.08
**Subject07**	0.21	**0.56**	0.39	0.23	0.47	0.41	0.5
**Subject08**	0.26	**0.58**	0.43	0.31	0.51	0.3	0.32
**Subject09**	0.27	0.3	0.28	0.25	0.5	**0.54**	0.19
**Subject10**	0.16	0.08	0.18	0.17	**0.57**	0.41	0.37
**Subject11**	0.09	0.13	0.01	0	**0.44**	0.21	0.27
**Subject12**	0.14	0.29	0.21	0.18	**0.58**	0.55	0.19
**Subject13**	0.21	0.26	0.19	0.15	**0.39**	0.19	0.18
**Subject14**	0.36	**0.44**	0.36	0.15	0.35	0.12	0.29
**Subject15**	0.13	0.23	0.21	0.2	**0.48**	0.28	0.31
**Subject16**	0.13	0.5	**0.57**	0.12	0.3	0.16	0.07
**Subject17**	0.15	**0.29**	0.06	0	0.26	0.1	0.12
**Subject18**	0.06	0.27	0.19	0.19	**0.63**	0.21	0.07
**Subject19**	0.24	0.19	0.08	0	**0.4**	0.23	0.15
**Subject20**	0.08	0.44	0.55	0.65	**0.77**	0.65	0.65
**Subject21**	0.32	0.42	0.42	0.26	**0.6**	0.49	0.46
**Subject22**	0.23	**0.4**	0.33	0.31	0.35	0.27	0.27
**Subject23**	0.11	0.27	0.37	0.39	**0.56**	0.27	0.22
**Subject24**	0.11	0.05	0.02	0	0.17	0.07	**0.23**
**Subject25**	0.26	0.61	0.6	0.57	**0.71**	0.64	0.64
**Subject26**	0.25	0.27	0.18	0	**0.38**	0.12	0.11
**Subject27**	0.32	0.29	0.44	0	**0.58**	0.05	0.12
**Subject28**	0.14	0.4	0.23	0.21	**0.44**	0.23	0.2
**Subject29**	0.4	0.32	0.29	0.19	**0.47**	0.37	0.36
**Subject30**	0.18	0.29	0.27	0.19	**0.37**	0.3	0.24
**Subject31**	0.17	0.51	0.58	0.56	**0.61**	0.57	0.57
**Averaged case**	0.57	0.71	**0.78**	0.66	0.71	0.58	0.51
**Mean score**	0.209	0.363	0.358	0.271	**0.51**	0.366	0.309

*Note:* The bolded result in each row presents the highest similarity score which calculated between the results of T‐SWCGL with different window length and KELLER's result. Abbreviations: KELLER, kernel‐reweighted logistic regres; T‐SWCGL, tapered sliding window graphical lasso; s, seconds.

For a better comparison, we focused on the mean dynamic pattern of regions involved in the default mode network (DMN) yielded by different methods for the averaged case. The names of the ROIs in the Harvard‐oxford atlas and their abbreviations are included in Table S7. These ROIs are selected in association with seven resting state networks of functionally coupled parcellated regions across the cerebral cortex (Yeo et al., 2011) as well as subcortical regions including amygdala, hippocampus, and para‐hippocampal regions. In Figure 7, we illustrated the dynamic connections in DMN yielded by different methods. We also listed the common dynamically connected ROI pairs identified by KELLER and T‐SWCGL in Table 5. Results showed that most of the commonly identified dynamic connections in DMN were between subcortical, temporal, and frontal regions. On the other hand, the list of dynamic connections in DMN identified only by specific methods are presented in Table 6. Most of those dynamic connections identified only by KELLER are located between posterior and anterior regions of DMN while T‐SWCGL identified connections between temporal and anterior region of DMN.

**TABLE 5:** Common dynamic connections in DMN identified by KELLER and T‐SWCGL with different window length (w = 40, 60, and 100 s).

	dFC connections in DMN
Common dFC connection in DMN between KELLER and T‐SWCGL (w = 40, 60, and 100 s)	Hippocampus (R), superior frontal gyrus (R) Hippocampus (R), anterior‐middle temporal gyrus(R) Hippocampus (R), Paracingulate gyrus (L) Hippocampus (R), hippocampus (L) Hippocampus (R), anterior‐cingulate gyrus (L) Anterior‐superior temporal gyrus(R), posterior‐middle temporal gyrus (L) Anterior‐superior temporal gyrus(R), frontal medial cortex (R) Frontal medial cortex (R), inferior frontal gyrus(R) Temporal pole (L), temporal pole (R) Temporal pole (L), posterior‐parahippocampal gyrus(L) Posterior‐parahippocampal gyrus (R)′, posterior‐superior temporal gyrus (L) Posterior‐parahippocampal gyrus (R)′, posterior‐superior temporal gyrus (R) Anterior‐middle temporal gyrus (L), posterior‐cingulate gyrus (L) Anterior‐middle temporal gyrus (R)′,frontal orbital cortex (L) Precuneous cortex (L), posterior‐middle temporal gyrus(R) Amygdala (R), posterior‐parahippocampal gyrus(L)
Common dFC connection DMN only between KELLER and T‐SWCGL (w = 40 s)	Hippocampus (R), superior frontal gyrus (L) Hippocampus (R), temporal pole (R) Hippocampus (R), angular gyrus (L) Hippocampus (R), anterior‐parahippocampal gyrus (R) Hippocampus (R), posterior‐parahippocampal gyrus (R) Posterior‐middle temporal gyrus (L), middle frontal gyrus (L) Temporooccipital‐middle temporal gyrus (L), amygdala (L) Angular gyrus (R), anterior‐ cingulate gyrus (L) Anterior‐parahippocampal gyrus (R), precuneous cortex (R)
Common dFC connection DMN only between KELLER and T‐SWCGL (w = 60 s)	Hippocampus (R), temporal pole (R) Posterior‐middle temporal gyrus (L), middle frontal gyrus (L) Inferior frontal gyrus (R), hippocampus (L) Anterior‐superior temporal gyrus (L), temporooccipital‐middle temporal gyrus(R) Temporooccipital‐middle temporal gyrus (L), amygdala (L) Angular gyrus (R), anterior‐cingulate gyrus (L) Anterior‐parahippocampal gyrus(R), precuneous cortex (R)
Common dFC connection DMN only between KELLER and T‐SWCGL (w = 100 s)	Inferior frontal gyrus (R), hippocampus (L) Inferior frontal gyrus (R), paracingulate gyrus (L) Posterior parahippocampal gyrus (R), anterior‐superior temporal gyrus (L)

Abbreviations: dFC, dynamic functional connectivity; DMN, default mode network; KELLER, kernel‐reweighted logistic regression; T‐SWCGL, tapered sliding window graphical lasso; L, left hemisphere; R, right hemisphere; s, seconds.

**TABLE 6:** Specific dynamic connection identified in DMN only by KELLER or T‐SWCGL with different window length (w = 40, 60, and 100 s).

dFC connections estimated only by KELLER	dFC connections estimated only by T‐SWCGL	W (s)
Inferior frontal gyrus (R), posterior‐superior temporal gyrus (R) Frontal medial cortex (R), posterior‐cingulate gyrus (R) Frontal medial cortex (R), precuneous cortex (R) Frontal medial cortex (R), anterior‐parahippocampal Gyrus (L) Posterior‐cingulate gyrus (L), frontal medial cortex (L) Posterior‐cingulate gyrus (L), precuneous cortex (L) Precuneous cortex (L), middle frontal gyrus (L)	Hippocampus (L), anterior‐parahippocampal gyrus (R) Hippocampus (R), anterior‐cingulate gyrus (R) Frontal medial cortex (L), anterior‐middle temporal gyrus (L) Posterior‐middle temporal gyrus (L), middle frontal gyrus (R) Temporal pole (L), anterior‐superior temporal gyrus (L) Anterior‐middle temporal gyrus (R), anterior‐cingulate gyrus (L) Anterior‐cingulate gyrus (L), posterior‐cingulate gyrus (L) Anterior‐middle temporal gyrus (L), posterior‐middle temporal gyrus (L) Anterior‐parahippocampal gyrus (L), paracingulate gyrus (R) Paracingulate gyrus (L), inferior frontal gyrus (R)	40, 60, and 100 40 and 60 40 and 60 40 40 40 40 40 60 and 100 100

Abbreviations: dFC, dynamic functional connectivity; KELLER, kernel‐reweighted logistic regression; T‐SWCGL, tapered sliding window graphical lasso; L, left hemisphere; R, right hemisphere; s, seconds.

## 5 DISCUSSION

### 5.1 Overview of the current study

The main goal of this study was to improve the power of detecting dynamic connections in estimated dFC by conventional methods. Thus, we introduced a framework developed in the gene regulatory studies, the KELLER algorithm (Song et al., 2009), and utilized it in the dFC network studies. KELLER allowed for the retrieval of the dFC pattern of brain networks in terms of the network's structure and modulation over time.

A series of simulation studies were done to measure the capability of KELLER in retrieving the underlying structure of dFC network as well as the power of KELLER in detecting dynamic connections in comparison with T‐SWCGL and SWCGL. We also employed the proposed method in estimating whole brain dFC networks from rs‐fMRI data of 31 healthy subjects to detect statistically significant dynamically connected brain region pairs. To achieve this, we performed proper statistical tests on the *SD* of dFC time series as a test statistic measure using appropriate surrogate data.

We demonstrated the following key results via simulation studies: (a) KELLER estimates dFC networks more accurately than T‐SWCGL and SWCGL and because of its more accurate estimation procedure, the detectability power of dynamic connections in KELLER is also higher than the other methods; (b) increasing/decreasing of window size in SWCGL and T‐SWCGL does not have any significant effect on their performance; (c) increasing the number of nodes and the complexity of network topology structures in the simulation studies affects the performance of all algorithms in terms of both the accuracy of estimated dFC networks as well as the power of dynamic connections detection.

The experimental findings illustrated that KELLER and T‐SWCGL, with different window lengths from 20 to 140 s, detect dynamic patterns in single subject rs‐fMRI data as well as in the averaged case. However, findings of KELLER and T‐SWCGL with window length of 100 s showed different patterns. In both obtained results by KELLER and T‐SWCGL with window length of 100 s, the number of significant dynamic connection for individuals are more than those of T‐SWCGL with window lengths of 20, 40, 60, 80, 120, and 140 s. Using either approach, averaging across all subjects increases significant dynamic connections substantially. This finding is in line with a previous study (Hindriks et al., 2016) and confirms that averaging across all subjects increases statistical power of null hypothesis testing in detecting dynamic connections which could be a result of the number of subjects used for averaging. Moreover, results revealed that the mean estimated dFC matrix of statistically significant dynamic connections by T‐SWCGL with window length of 100 s among different window lengths has the highest similarity to the KELLER method. This finding is consistent with previous studies (Leonardi & Van De Ville, 2015; Zalesky & Breakspear, 2015) suggesting that appropriate window length for SWC studies should be equal or larger than 100 s. Interestingly, in the averaged case, the highest similarity between KELLER and T‐SWCGL was achieved for a window length of 60 s, followed by window lengths of 40 and 100 s. We then focused on DMN dynamic pattern based on the mean dFC pattern of the averaged case by the KELLER and T‐SWCGL (w = 40, 60, and 100 s) methods in order to allow us to go deeper for a precise comparison. Results revealed that most of the common identified dynamic connections in DMN are between subcortical, temporal and frontal regions. On the other hand, most of those dynamic connections identified only by KELLER method are located between posterior and anterior regions of DMN while T‐SWCGL method identified connections between temporal and anterior regions of DMN.

### 5.2 Previous studies in assessing dFC in rs‐fMRI by using SWC technique and comparison with the present study

The effect of different sliding window parameters such as window type, length, and step, as well as different FC metrics on the detection of dynamic connections or brain states have been investigated (Hindriks et al., 2016; Savva et al., 2019; Shakil et al., 2016). In (Shakil et al., 2016), segmented real BOLD time series were mixed to form a simulated setting where the switching between brain states were known. Their findings implied that window length and size affected the identification of brain state switching (Shakil et al., 2016). Similar to our findings on experimental fMRI data showed that window length influenced the results of T‐SWCGL method. In another study, they tried to answer similar questions by applying different FC metrics to estimated dFC network and using experimental rs‐fMRI data in two separate groups for test–retest validation (Savva et al., 2019). The authors showed that the obtained results based on mutual information and variation of information with a window length larger than 120 s yielded the most consistent results by applying test–retest analysis. Moreover, in (Hindriks et al., 2016) it was claimed that it was impossible to detect dynamic connections by using SWC in individual sessions through simulation studies and this claim was validated using both rs‐fMRI data of humans and macaques (Hindriks et al., 2016). Recently, the possible impact of global signal regression on the estimation of dynamic connection and brain states was evaluated by H. Xu et al., (2018). Results showed that the impact of global signal regression on dFC was temporally modulated by the mean global signal magnitude across windows and authors suggested that global signal regression should be applied to SWC analyses with caution. In the present study, we evaluated the impact of global signal regression on KELLER and T‐SWCGL with window length of 100 s (Figures S1 and S2, respectively). We also investigated the influence of global signal regression on the mean dynamic pattern of DMN for the averaged case, estimated by KELLER and T‐SWCGL with window length of 100 s. The results showed a considerable change when considering global signal regression. In fact, the number of dynamic connections decreased considerably in both methods when considering global signal regression, because global signal increases the dependencies among brain regions (Figure S3). It is noteworthy that most of the previous dFC studies including (Hindriks et al., 2016; Shakil et al., 2016) have not considered the impact of global signal regression on their findings.

In the present study, we used KELLER (Song et al., 2009) to estimate dFC networks at each time point of BOLD signal measurement. The important feature of KELLER is that it considers multivariate dependencies between brain regions to estimate dFC networks through estimating functional pattern of each brain region spatially and temporally. SWC‐based methods only capture bivariate linear association between brain regions. Additionally, KELLER also has the potential to define a proper window length to extract dFC time series, utilizing AIC as a model selection approach. In this study, for KELLER, we defined a range of values (from 10 to 150 with 10 s steps) for the window length parameter, *h*, Then, we employed an extensive grid‐search in the given range of the parameters *h* and *δ*. We used AIC to estimate the in‐sample prediction error for each choice of parameters *h* and *δ*, allowing for a direct comparison across different values of each parameter. Finally, a pair of parameters that minimizes AIC is chosen as the optimal values. Moreover, the structure of dFC networks is automatically estimated at each time point because of the ℓ1‐penalized term in the loss function optimized by KELLER which in turn yields a sparse network effectively.

To evaluate the performance of the proposed approach, we compared it with T‐SWCGL and SWCGL in terms of their abilities to recover structure of dFC networks over time as well as detecting dynamically connected brain region pairs. T‐SWCGL and SWCGL model dFC networks using correlation analysis at the *i* ^th^ window of observations. For a fair comparison, we applied graphical lasso to consider the sparsity of the estimated dFC networks. Moreover, to minimize the effect of window length in the capability of the SWC‐based methods, we used various window lengths, from 20 to 140 s in 20 s steps. The simulation results suggested that KELLER was superior to T‐SWCGL and SWCGL in terms of the mean F_1_ score. It is notable to mention that the capability of considering multivariate dependencies between brain regions in KELLER results in the accurate estimation of dFC networks, which in turn may be the main reason of improving the detection of dynamic connections in the estimated dFC network by KELLER. As expected, modeling multivariate dependencies in estimating dFC networks increased the number of dynamic connections.

### 5.3 “The higher number of detected dynamic connections, the more statistical power of method to estimate dFC matrices,” is this correct?

Answering this question is not straightforward. Actually, if there were a ground truth for dynamic behavior of brain region pairs, then this statement could be evaluated. In fact, the absence of ground truth in human brain network analysis warns us that the higher number of dynamically connected regions detected should not be interpreted as higher statistical power of a specific method in estimating dFC matrices (Savva et al., 2019; Shakil et al., 2016). However, to determine which method is the most appropriate to be applied in dFC analysis, there are two approaches: (a) designing simulation studies that provide ground truth for evaluation; and (b) comparing the results obtained on real data with those reported in the literature. Since there is no comprehensive study that investigated and reported dynamically connected brain region pairs in the whole brain, we only focused on the detected dynamically connected regions with posterior cingulate cortex (PCC) in DMN and compared the results with those of the previous studies (Chang & Glover, 2010; Savva et al., 2019). Specifically, in Savva et al., (2019), the presence of dynamic connections between brain regions comprising DMN was examined by employing various window lengths and FC metrics. They found that PCC and bilateral parietal lobes were involved in most dynamic connections as the hub regions of dorsal and ventral DMN, respectively (Savva et al., 2019). Moreover, in Chang & Glover, (2010), dynamic connections between PCC and those brain regions that are correlated or anti‐correlated with PCC were examined, utilizing wavelet transform. The authors found that phase coupling between these regions and PCC were dynamic (Chang & Glover, 2010). In Table 5, the results are shown for the common dynamic connections detected by KELLER and T‐SWCGL (w = 40, 60 and 100 s), focusing on DMN (Shirer, Ryali, Rykhlevskaia, Menon, & Greicius, 2012). Most of those commonly detected dynamic connections are located between subcortical, temporal and frontal regions of DMN. On the other hand, in Table 6, we reported specific dynamic connections detected only by KELLER or T‐SWCGL. For KELLER, these connections are specially located at dorsal DMN connection pairs (PCC‐frontal cortex, PCC‐precuneus, and precuneus‐frontal cortex) which were commonly detected in the previous studies (Chang & Glover, 2010; Savva et al., 2019). As can be seen in Table 6, most of the mentioned connection pairs detected by previous studies are detected as dynamically connected by utilizing KELLER. On the other hand, SWC‐based method detected only one dynamic connection in dorsal DMN between PCC and frontal cortex, suggesting that Pearson correlation coefficient has less sensitivity to detect dFC. This finding is consistent with previous studies (Hindriks et al., 2016; Savva et al., 2019).

### 5.4 Study limitations and future work

We mentioned some limitations of SWC‐based methods in this study and introduced KELLER to overcome them. However, KELLER also has its own limitations. For instance, it is very time consuming due to computational complexity of the optimization algorithm and the model selection approach involved in KELLER. This is especially true when the number of involved brain regions is large. On the other hand, SWC‐based methods are time efficient in estimating dFC networks. However, since these methods have low sensitivity in detecting previously reported dynamic connections (Tables 5 and 6), developing a powerful method to estimate dFC time series with high accuracy is of critical importance.

Assessment of the estimated dFC networks to detect statistically significant dynamically connected brain region pairs is highly suggested for future work in this vibrant area of neuroimaging research. Future work can also apply KELLER for estimating dFC from rs‐fMRI data of neurodegenerative and neuropsychiatric disorders to investigate the abnormal dynamic connectivity patterns and compare their findings with those of the conventional methods.

## 6 CONCLUSION

In this study, a comprehensive analysis of dFC in the whole brain using rs‐fMRI data were performed by employing the newly introduced KELLER method considering multivariate dependencies between brain regions to estimate dFC network. In order to evaluate the proposed dFC estimation method in comparison with SWC, a series of simulation studies was implemented providing ground truth, and then a hypothesis testing framework was applied to detect dynamically connected region pairs. The simulation results showed that KELLER outperformed T‐SWCGL and SWCGL approaches in retrieving dFC pattern of brain networks in terms of the network's structure and modulation over time as well as in detecting dynamically connected brain regions. Experimental results showed that KELLER was able to detect previously reported dynamically connected brain region pairs within DMN. Overall, dFC network analysis has a promising capability for a better understanding of the brain as well as contributing to the development of new biomarkers for diagnosis or prognosis of mental disorders. However, statistical assessment of the estimated dFC should be done as a primary step to ensure that the detected dFC patterns are due to the dynamic nature of the cognitive process or resting state condition in the brain.

## CONFLICT OF INTERESTS

The authors declare no potential conflict of interest.

## Supporting information


**Appendix** S1: Supplemental Information

## Data Availability

Data used in this study were obtained from 1000 Functional Connectomes Project (ftp://www.nitrc.org/fcon_1000/htdocs/SaintLouis.tar), a publicly available database.
