# Supplementary material for: Improved dynamic connection detection power in estimated dynamic functional connectivity considering multivariate dependencies between brain regions
Source: Hum Brain Mapp. 2020 Jul 9;41(15):4264–87. doi: 10.1002/hbm.25124 (PMC7502846; doi:10.1002/hbm.25124)
Supplement: Supplementary file 1 — Appendix S1: Supplemental Information [file HBM-41-4264-s001.docx]

**Supplemental Information**

**Improved Dynamic Connection Detection Power in Estimated Dynamic Functional Connectivity Considering Multivariate Dependencies between Brain Regions**

**SI-1. Projected Gradient (PG) Algorithm**

$$\boldsymbol{Algorithm}\boldsymbol{1}$$

$\boldsymbol{Input}\boldsymbol{:}\boldsymbol{T}\boldsymbol{ime series}\left( \boldsymbol{y}^{\left( \boldsymbol{1} \right)}\boldsymbol{,\ldots,}\boldsymbol{y}^{\left( \boldsymbol{t} \right)}\boldsymbol{,\ldots,}\boldsymbol{y}^{\left( \boldsymbol{T} \right)} \right) \boldsymbol{where}\boldsymbol{y}^{\left( \boldsymbol{t} \right)}\boldsymbol{=}\left( \boldsymbol{y}_{\boldsymbol{1}}^{\left( \boldsymbol{t} \right)}\boldsymbol{,\ldots,}\boldsymbol{y}_{\boldsymbol{p}}^{\left( \boldsymbol{t} \right)} \right)$ $\boldsymbol{an}\boldsymbol{d}\boldsymbol{upper bound}\boldsymbol{C}_{\boldsymbol{\delta}}$

$$\boldsymbol{Initialize}{\hat{\boldsymbol{\theta}}}_{\boldsymbol{-i}}^{\boldsymbol{(t)}}\boldsymbol{,}{\tilde{\boldsymbol{\theta}}}_{\boldsymbol{-i}}^{\boldsymbol{(t)}}\boldsymbol{,set \alpha=0.1, \varepsilon=}\boldsymbol{10}^{\boldsymbol{-6}}\boldsymbol{,\sigma=}\boldsymbol{10}^{\boldsymbol{-2}}$$

$$\boldsymbol{for t=1:T}$$

$$\boldsymbol{for i=1:p}$$

$$\boldsymbol{while}\left\| {\hat{\boldsymbol{\theta}}}_{\boldsymbol{-}\boldsymbol{i}}^{\left( \boldsymbol{t} \right)}\boldsymbol{-}{\tilde{\boldsymbol{\theta}}}_{\boldsymbol{-}\boldsymbol{i}}^{\left( \boldsymbol{t} \right)} \right\|\boldsymbol{\geq}\boldsymbol{\varepsilon} \boldsymbol{do}$$

$${\hat{\boldsymbol{\theta}}}_{\boldsymbol{-i}}^{\boldsymbol{(t)}}\boldsymbol{\leftarrow}{\tilde{\boldsymbol{\theta}}}_{\boldsymbol{-i}}^{\left( \boldsymbol{t} \right)}\boldsymbol{, \eta\leftarrow1.0}$$

$\boldsymbol{while (L}\left( {\tilde{\boldsymbol{\theta}}}_{\boldsymbol{-i}}^{\left( \boldsymbol{t} \right)} \right)\boldsymbol{-L}\left( {\hat{\boldsymbol{\theta}}}_{\boldsymbol{-i}}^{\left( \boldsymbol{t} \right)} \right)\boldsymbol{)\geq\sigma}\boldsymbol{\nabla}^{\left( \boldsymbol{t} \right)}\boldsymbol{(}{\tilde{\boldsymbol{\theta}}}_{\boldsymbol{-i}}^{\left( \boldsymbol{t} \right)}\boldsymbol{-}{\hat{\boldsymbol{\theta}}}_{\boldsymbol{-i}}^{\left( \boldsymbol{t} \right)}\boldsymbol{)}$ ***do***

$${\tilde{\boldsymbol{\theta}}}_{\boldsymbol{-i}}^{\left( \boldsymbol{t} \right)}\boldsymbol{\leftarrow}\boldsymbol{\Pi}_{\boldsymbol{\Omega}}\left( \boldsymbol{\theta}_{\boldsymbol{-i}}^{\boldsymbol{(t)}}\boldsymbol{-\eta}\boldsymbol{\nabla}^{\boldsymbol{(t)}} \right)\boldsymbol{, \eta\leftarrow\alpha\eta}$$

$$\boldsymbol{end}$$

$$\boldsymbol{end}$$

$${\hat{\boldsymbol{\theta}}}_{\boldsymbol{-i}}^{\left( \boldsymbol{t} \right)}\boldsymbol{\leftarrow}{\tilde{\boldsymbol{\theta}}}_{\boldsymbol{-i}}^{\left( \boldsymbol{t} \right)}$$

$$\boldsymbol{return}{\hat{\boldsymbol{\theta}}}_{\boldsymbol{-i}}^{\boldsymbol{(t)}}$$

$$\boldsymbol{end}$$

$$\boldsymbol{return} {\hat{\boldsymbol{\theta}}}^{\boldsymbol{(t)}}$$

$$\boldsymbol{end}$$

SI-Table 1- Performance of different three methods in estimating dynamic functional connectivity networks in terms of F1 score as the number of nodes was increased from 5 to 70 with two different simulated network topology structures: A) Erdős–Rényi random network; B) Scale-free network. In the case of T-SWCGL and SWCGL methods, the window length is set to 100 sec.

| **Number of Nodes**  **Different network**  **structure and methods** | | **5** | | **10** | | **20** | | **30** | | **40** | | **50** | | **60** | | **70** | |
| --- | --- | --- | --- | --- | --- | --- | --- | --- | --- | --- | --- | --- | --- | --- | --- | --- | --- |
|  |  | $\boldsymbol{mean\pm SD}$ | **p-value** | $\boldsymbol{mean\pm SD}$ | **p-value** | $\boldsymbol{mean\pm SD}$ | **p-value** | $\boldsymbol{mean\pm SD}$ | **p-value** | $\boldsymbol{mean\pm SD}$ | **p-value** | $\boldsymbol{mean\pm SD}$ | **p-value** | $\boldsymbol{mean\pm SD}$ | **p-value** | $\boldsymbol{mean\pm SD}$ | **p-value** |
| **Erdos - Renyi random network** | KELLER vs. T-SWCGL | 0.94 ± 0.01 | <0.001 | 0.91 ± 0.04 | 0.037 | 0.88 ± 0.05 | <0.001 | 0.87 ± 0.05 | <0.001 | 0.85 ± 0.07 | <0.001 | 0.80 ± 0.07 | <0.001 | 0.79 ± 0.06 | <0.001 | 0.78 ± 0.08 | 0.001 |
|  |  | 0.76 ± 0.01 |  | 0.71 ± 0.1 |  | 0.64 ± 0.09 |  | 0.59 ± 0.08 |  | 0.57 ± 0.09 |  | 0.56 ± 0.07 |  | 0.54 ± 0.1 |  | 0.54 ± 0.1 |  |
|  | KELLER vs. SWCGL | 0.94 ± 0.01 | <0.001 | 0.91 ± 0.04 | <0.001 | 0.88 ± 0.05 | <0.001 | 0.87 ± 0.05 | <0.001 | 0.85 ± 0.07 | <0.001 | 0.80 ± 0.07 | <0.001 | 0.79 ± 0.06 | <0.001 | 0.78 ± 0.08 | <0.001 |
|  |  | 0.71 ± 0.02 |  | 0.62 ± 0.11 |  | 0.57 ± 0.1 |  | 0.51 ± 0.08 |  | 0.49 ± 0.09 |  | 0.48 ± 0.08 |  | 0.47 ± 0.12 |  | 0.47 ± 0.11 |  |
|  | T-SWCGL vs. SWCGL | 0.76 ± 0.01 | <0.001 | 0.71 ± 0.1 | 0.002 | 0.64 ± 0.09 | 0.01 | 0.59 ± 0.08 | <0.001 | 0.57 ± 0.09 | <0.001 | 0.56 ± 0.07 | <0.001 | 0.54 ± 0.1 | 0.066 | 0.54 ± 0.1 | 0.121 |
|  |  | 0.71 ± 0.02 |  | 0.62 ± 0.11 |  | 0.57 ± 0.1 |  | 0.51 ± 0.08 |  | 0.49 ± 0.09 |  | 0.48 ± 0.08 |  | 0.47 ± 0.12 |  | 0.47 ± 0.11 |  |
| **Scale - Free random network** | KELLER vs. T-SWCGL | 0.82 ± 0.03 | <0.001 | 0.76 ± 0.04 | 0.081 | 0.71 ± 0.04 | <0.001 | 0.71 ± 0.03 | <0.001 | 0.70 ± 0.05 | <0.001 | 0.70 ± 0.06 | <0.001 | 0.70 ± 0.04 | <0.001 | 0.69 ± 0.06 | <0.001 |
|  |  | 0.69 ± 0.05 |  | 0.65 ± 0.07 |  | 0.58 ± 0.07 |  | 0.56 ± 0.06 |  | 0.54 ± 0.06 |  | 0.54 ± 0.05 |  | 0.52 ± 0.07 |  | 0.51 ± 0.08 |  |
|  | KELLER vs. SWCGL | 0.82 ± 0.03 | <0.001 | 0.76 ± 0.04 | <0.001 | 0.71 ± 0.04 | <0.001 | 0.71 ± 0.03 | <0.001 | 0.70 ± 0.05 | <0.001 | 0.70 ± 0.06 | <0.001 | 0.70 ± 0.04 | <0.001 | 0.69 ± 0.06 | 0.002 |
|  |  | 0.59 ± 0.05 |  | 0.55 ± 0.08 |  | 0.53 ± 0.08 |  | 0.53 ± 0.06 |  | 0.51 ± 0.06 |  | 0.5 ± 0.06 |  | 0.48± 0.09 |  | 0.48± 0.08 |  |
|  | T-SWCGL vs. SWCGL | 0.69 ± 0.05 | <0.001 | 0.65 ± 0.07 | <0.001 | 0.58 ± 0.07 | <0.001 | 0.56 ± 0.06 | <0.001 | 0.54 ± 0.06 | 0.066 | 0.54 ± 0.05 | <0.001 | 0.52 ± 0.07 | 0.001 | 0.51 ± 0.08 | 0.325 |
|  |  | 0.59 ± 0.05 |  | 0.55 ± 0.08 |  | 0.53 ± 0.08 |  | 0.53 ± 0.06 |  | 0.51 ± 0.06 |  | 0.5 ± 0.06 |  | 0.48± 0.09 |  | 0.48± 0.08 |  |

SI-Table 2- Averaged Confusion matrix over 100 runs for three different methods in estimating dynamic connections as the number of nodes was increased from 5 to 70 with Erdős–Rényi random network topology structures. In the case of T-SWCGL and SWCGL methods, the window length is set to 100 sec.

| **RANDOM**  **NETWORK** | | **5** | | **10** | | **20** | | **30** | | **40** | | **50** | | **60** | | **70** | |
| --- | --- | --- | --- | --- | --- | --- | --- | --- | --- | --- | --- | --- | --- | --- | --- | --- | --- |
|  |  | **P** | **N** | **P** | **N** | **P** | **N** | **P** | **N** | **P** | **N** | **P** | **N** | **P** | **N** | **P** | **N** |
| **KELLER** | **P** | 1916 | 168 | 7141 | 1008 | 28750 | 7196 | 62863 | 16733 | 108210 | 29811 | 159800 | 49573 | 228510 | 79158 | 319570 | 102800 |
|  | **N** | 76 | 2865 | 407 | 11544 | 625 | 43829 | 2019 | 99285 | 8299 | 175280 | 30117 | 263010 | 41872 | 374060 | 77820 | 484710 |
| **T-SWCGL** | **P** | 1548 | 535 | 5993 | 3335 | 21551 | 16355 | 44487 | 41286 | 77531 | 77966 | 133310 | 152770 | 178320 | 211073 | 240360 | 251410 |
|  | **N** | 444 | 2498 | 1555 | 9217 | 7824 | 34670 | 20395 | 74732 | 38973 | 127130 | 56610 | 159810 | 92067 | 242140 | 157030 | 336100 |
| **SWCGL** | **P** | 1519 | 766 | 5215 | 4056 | 18613 | 17287 | 39687 | 50979 | 66485 | 88283 | 109750 | 157070 | 159640 | 248540 | 204780 | 269120 |
|  | **N** | 473 | 2267 | 2334 | 8495 | 10762 | 33738 | 25196 | 65038 | 50022 | 116810 | 80170 | 155510 | 110740 | 204680 | 192620 | 318380 |

SI-Table 3- Averaged Confusion matrix over 100 runs for three different methods in estimating dynamic connections as the number of nodes was increased from 5 to 70 with Scale-free network topology structures. In the case of T-SWCGL and SWCGL methods, the window length is set to 100 sec.

| **SCALE-FREE**  **NETWORK** | | **5** | | **10** | | **20** | | **30** | | **40** | | **50** | | **60** | | **70** | |
| --- | --- | --- | --- | --- | --- | --- | --- | --- | --- | --- | --- | --- | --- | --- | --- | --- | --- |
|  |  | **P** | **N** | **P** | **N** | **P** | **N** | **P** | **N** | **P** | **N** | **P** | **N** | **P** | **N** | **P** | **N** |
| **KELLER** | **P** | 1891 | 614 | 6802 | 3169 | 26456 | 12686 | 58156 | 30745 | 100860 | 56249 | 157191 | 86088 | 227720 | 122980 | 309210 | 178410 |
|  | **N** | 216 | 2304 | 1109 | 9020 | 8920 | 32338 | 16779 | 75220 | 30001 | 134490 | 48609 | 210612 | 72020 | 300880 | 98986 | 398300 |
| **T-SWCGL** | **P** | 1561 | 858 | 5592 | 3688 | 22657 | 20115 | 49758 | 52914 | 80978 | 87879 | 114810 | 104494 | 172120 | 190290 | 235730 | 279780 |
|  | **N** | 546 | 2060 | 2318 | 8502 | 12718 | 24910 | 25177 | 53051 | 49883 | 102860 | 90986 | 192220 | 127620 | 233570 | 172460 | 296930 |
| **SWCGL** | **P** | 1375 | 1177 | 4723 | 4542 | 20676 | 21947 | 46596 | 54187 | 75766 | 90298 | 106770 | 114334 | 165350 | 223850 | 221200 | 292240 |
|  | **N** | 732 | 1741 | 3188 | 7647 | 14699 | 23078 | 28339 | 51778 | 55096 | 100440 | 99026 | 182370 | 134390 | 200010 | 186980 | 284470 |

SI-Table 4- Estimated sparsity in the simulation study using random network as the structure of simulated network by KELLER, T-SWCGL and SWCGL while the number of nodes increased from 5 to 70.

| **RANDOME**  **Network** | **Number of Nodes** | **5** | **10** | **20** | **30** | **40** | **50** | **60** | **70** |
| --- | --- | --- | --- | --- | --- | --- | --- | --- | --- |
|  | **True Sparsity** | **0.6** | **0.63** | **0.64** | **0.64** | **0.64** | **0.62** | **0.63** | **0.6** |
| **Estimated sparsity** | KELLER | $0.59\pm0.08$ | $0.60\pm0.06$ | $0.55\pm0.03$ | $0.56\pm0.1$ | $0.57\pm0.09$ | $0.58\pm0.08$ | $0.58\pm0.08$ | $0.57\pm0.08$ |
|  | T-SWCGL | $0.59\pm0.12$ | $0.54\pm0.1$ | $0.53\pm0.15$ | $0.53\pm0.12$ | $0.52\pm0.11$ | $0.43\pm0.2$ | $0.46\pm0.19$ | $0.5\pm0.15$ |
|  | SWCGL | $0.55\pm0.1$ | $0.54\pm0.13$ | $0.55\pm0.11$ | $0.50\pm0.17$ | $0.52\pm0.15$ | $0.47\pm0.18$ | $0.44\pm0.16$ | $0.52\pm0.1$ |

SI-Table 5- Estimated sparsity in the simulation study using scale-free network as the structure of simulated network by KELLER, T-SWCGL and SWCGL while the number of nodes increased from 5 to 70.

| **SCALE-FREE**  **NETWORK** | **Number of nodes** | **5** | **10** | **20** | **30** | **40** | **50** | **60** | **70** |
| --- | --- | --- | --- | --- | --- | --- | --- | --- | --- |
|  | **True Sparsity** | **0.58** | **0.61** | **0.56** | **0.59** | **0.59** | **0.59** | **0.59** | **0.59** |
| **Estimated Sparsity** | **KELLER** | $0.5\pm0.11$ | $0.5\pm0.08$ | $0.51\pm0.06$ | $0.51\pm0.1$ | $0.51\pm0.11$ | $0.52\pm0.08$ | $0.52\pm0.11$ | $0.51\pm0.09$ |
|  | **T-SWCGL** | $0.52\pm0.1$ | $0.54\pm0.07$ | $0.47\pm0.1$ | $0.43\pm0.12$ | $0.48\pm0.14$ | $0.56\pm0.06$ | $0.5\pm0.12$ | $0.48\pm0.08$ |
|  | **SWCGL** | $0.49\pm0.1$ | $0.54\pm0.09$ | $0.47\pm0.12$ | $0.44\pm0.18$ | $0.48\pm0.15$ | $0.56\pm0.07$ | $0.46\pm0.16$ | $0.48\pm0.1$ |

SI-Table 6- Percentage of detected dynamic connections based on estimated dynamic functional connectivity networks by three different methods as the number of nodes was increased from 5 to 70 with two different simulated network topology structures: A) Erdős–Rényi random network; B) Scale-free network. In the case of T-SWCGL and SWCGL methods, the window length is set to 100 sec.

| **Number of Nodes**  **Different network**  **structure and methods** | | **5** | | **10** | | **20** | | **30** | | **40** | | **50** | | **60** | | **70** | |
| --- | --- | --- | --- | --- | --- | --- | --- | --- | --- | --- | --- | --- | --- | --- | --- | --- | --- |
|  |  | $\boldsymbol{mean\pm SD}$ | **p-value** | $\boldsymbol{mean\pm SD}$ | **p-value** | $\boldsymbol{mean\pm SD}$ | **p-value** | $\boldsymbol{mean\pm SD}$ | **p-value** | $\boldsymbol{mean\pm SD}$ | **p-value** | $\boldsymbol{mean\pm SD}$ | **p-value** | $\boldsymbol{mean\pm SD}$ | **p-value** | $\boldsymbol{mean\pm SD}$ | **p-value** |
| **Erdos - Renyi random network** | KELLER vs. T-SWCGL | 87.3 ± 6.4 | 0.001 | 84.6 ± 7.2 | <0.001 | 82.8 ± 8.34 | <0.001 | 80.9 ± 8.15 | <0.001 | 79.1 ± 9.18 | <0.001 | 75.4 ± 10.11 | <0.001 | 74.5 ± 8.78 | <0.001 | 73.6 ± 12.43 | <0.001 |
|  |  | 70.1 ± 8.3 |  | 66.6 ± 10.23 |  | 59.7 ± 11.67 |  | 55.5 ± 10.78 |  | 54 ± 12.34 |  | 52.2 ± 13.67 |  | 52.1 ± 14.21 |  | 51.3 ± 14.58 |  |
|  | KELLER vs. SWCGL | 87.3 ± 6.4 | <0.001 | 84.6 ± 7.2 | <0.001 | 82.8 ± 8.34 | <0.001 | 80.9 ± 8.15 | <0.001 | 79.1 ± 9.18 | <0.001 | 75.4 ± 10.11 | <0.001 | 74.5 ± 8.78 | <0.001 | 73.6 ± 12.43 | <0.001 |
|  |  | 58.5 ± 6.2 |  | 55.3 ± 8.67 |  | 50.9 ± 10.56 |  | 44.9 ± 11.04 |  | 43 ± 13.83 |  | 43 ± 13.83 |  | 42.7 ± 15.43 |  | 42 ± 15.65 |  |
|  | T-SWCGL vs. SWCGL | 70.1 ± 8.3 | 0.003 | 66.6 ± 10.23 | 0.002 | 59.7 ± 11.67 | 0.002 | 55.5 ± 10.78 | 0.001 | 54 ± 12.34 | 0.015 | 52.2 ± 13.67 | 0.024 | 52.1 ± 14.21 | 0.158 | 51.3 ± 14.58 | 0.036 |
|  |  | 58.5 ± 6.2 |  | 55.3 ± 8.67 |  | 50.9 ± 10.56 |  | 44.9 ± 11.04 |  | 43 ± 13.83 |  | 43 ± 13.83 |  | 42.7 ± 15.43 |  | 42 ± 15.65 |  |
| **Scale - Free random network** | KELLER vs. T-SWCGL | 78.7 ± 3.54 | <0.001 | 73.9 ± 5.03 | <0.001 | 68.6 ± 4.96 | 0.009 | 68 ± 5.56 | <0.001 | 65.6 ± 6.87 | <0.001 | 65.6 ± 7.45 | <0.001 | 65.6 ± 8.76 | <0.001 | 65 ± 8.71 | <0.001 |
|  |  | 63 ± 5.78 |  | 61 ± 7.65 |  | 54.4 ± 8.32 |  | 52.2 ± 8.98 |  | 50.6 ± 9.02 |  | 50.1 ± 9.54 |  | 48.8 ± 9.69 |  | 47.5 ± 10.15 |  |
|  | KELLER vs. SWCGL | 78.7 ± 3.54 | <0.001 | 73.9 ± 5.03 | <0.001 | 68.6 ± 4.96 | <0.001 | 68 ± 5.56 | <0.001 | 65.6 ± 6.87 | <0.001 | 65.6 ± 7.45 | <0.001 | 65.6 ± 8.76 | <0.001 | 65 ± 8.71 | <0.001 |
|  |  | 51.2 ± 6.96 |  | 49 ± 8.26 |  | 47.6 ± 9.1 |  | 46.4 ± 9.56 |  | 45.6 ± 10.32 |  | 44.9 ± 10.65 |  | 44 ± 11.14 |  | 43.5 ± 11.76 |  |
|  | T-SWCGL vs. SWCGL | 63 ± 5.78 | 0.001 | 61 ± 7.65 | 0.003 | 54.4 ± 8.32 | 0.011 | 52.2 ± 8.98 | 0.007 | 50.6 ± 9.02 | 0.164 | 50.1 ± 9.54 | 0.003 | 48.8 ± 9.69 | 0.006 | 47.5 ± 10.15 | 0.009 |
|  |  | 51.2 ± 6.96 |  | 49 ± 8.26 |  | 47.6 ± 9.1 |  | 46.4 ± 9.56 |  | 45.6 ± 10.32 |  | 44.9 ± 10.65 |  | 44 ± 11.14 |  | 43.5 ± 11.76 |  |

SI-Table 7- Bilateral Brain Regions involved in default mode network

| **Regions name in Harvard-Oxford Atlas** | **Abbreviation** |
| --- | --- |
| 1. Superior Frontal Gyrus 2. Middle Frontal Gyrus 3. Inferior Frontal Gyrus, pars triangularis 4. Temporal Pole 5. Superior Temporal Gyrus, anterior division 6. Superior Temporal Gyrus, posterior division 7. Middle Temporal Gyrus, anterior division 8. Middle Temporal Gyrus, posterior division 9. Middle Temporal Gyrus, temporooccipital part 10. Angular Gyrus 11. Frontal Medial Cortex 12. Paracingulate Gyrus 13. Cingulate Gyrus, posterior division 14. Precuneous Cortex 15. Frontal Orbital Cortex 16. Hippocampus 17. Amygdala 18. Parahippocampal Gyrus, posterior division 19. Parahippocampal Gyrus, anterior division 20. Cingulate Gyrus, anterior division | SFG  MFG  IFG  TP  STG_a_  STG_p_  MTG_a_  MTG_p_  MTG_o_  AG  FMC  paraCG  PCC  Prec  FOC  Hip  Amy  paraHip_a_  paraHip_p_  ACC |


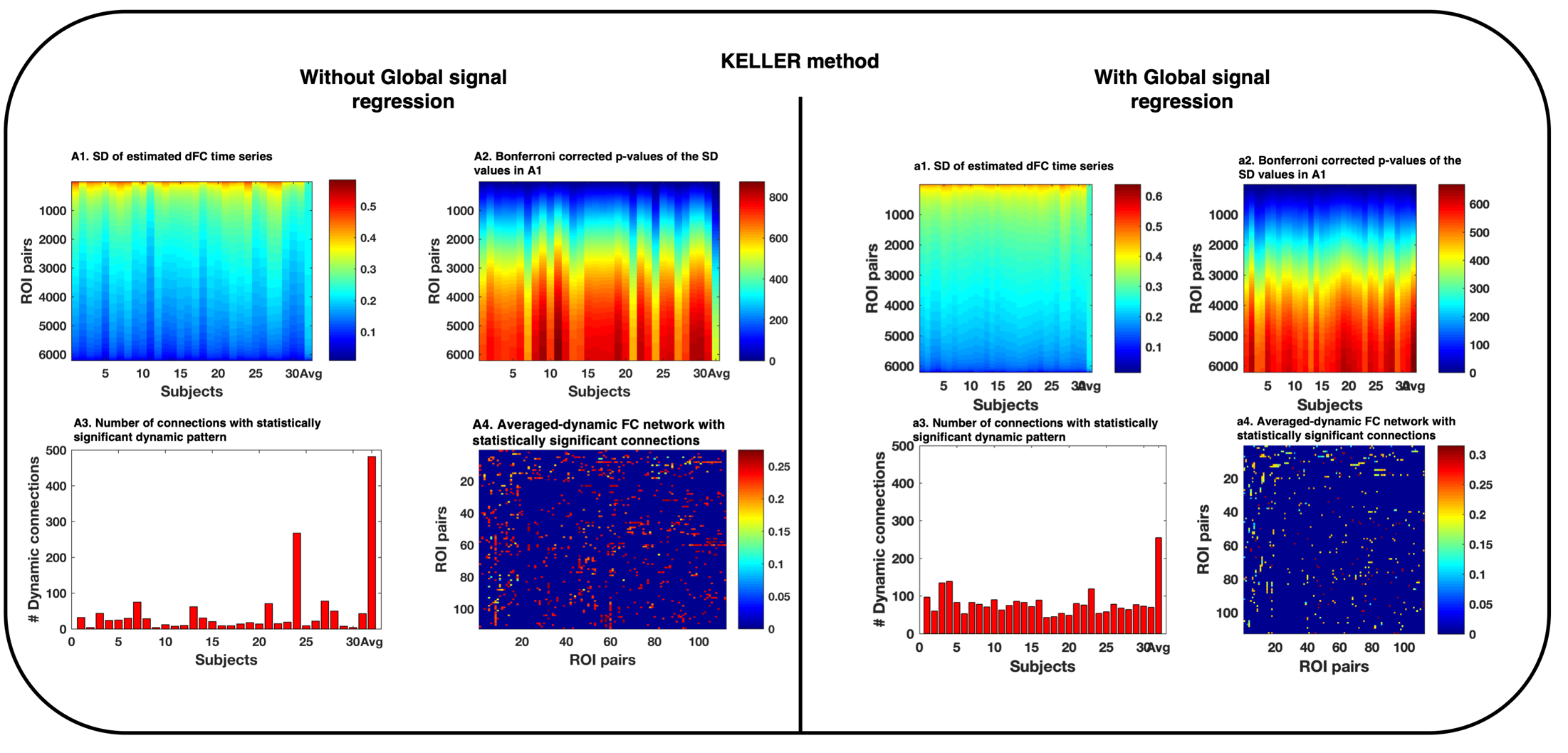


SI-Figure 1- The influence of global signal regression on the results of KELLER. The results showed that GSR considerably increased the dynamic detection power of single subjects, while dynamic detection power also was increased by subject-averaging of the measures.


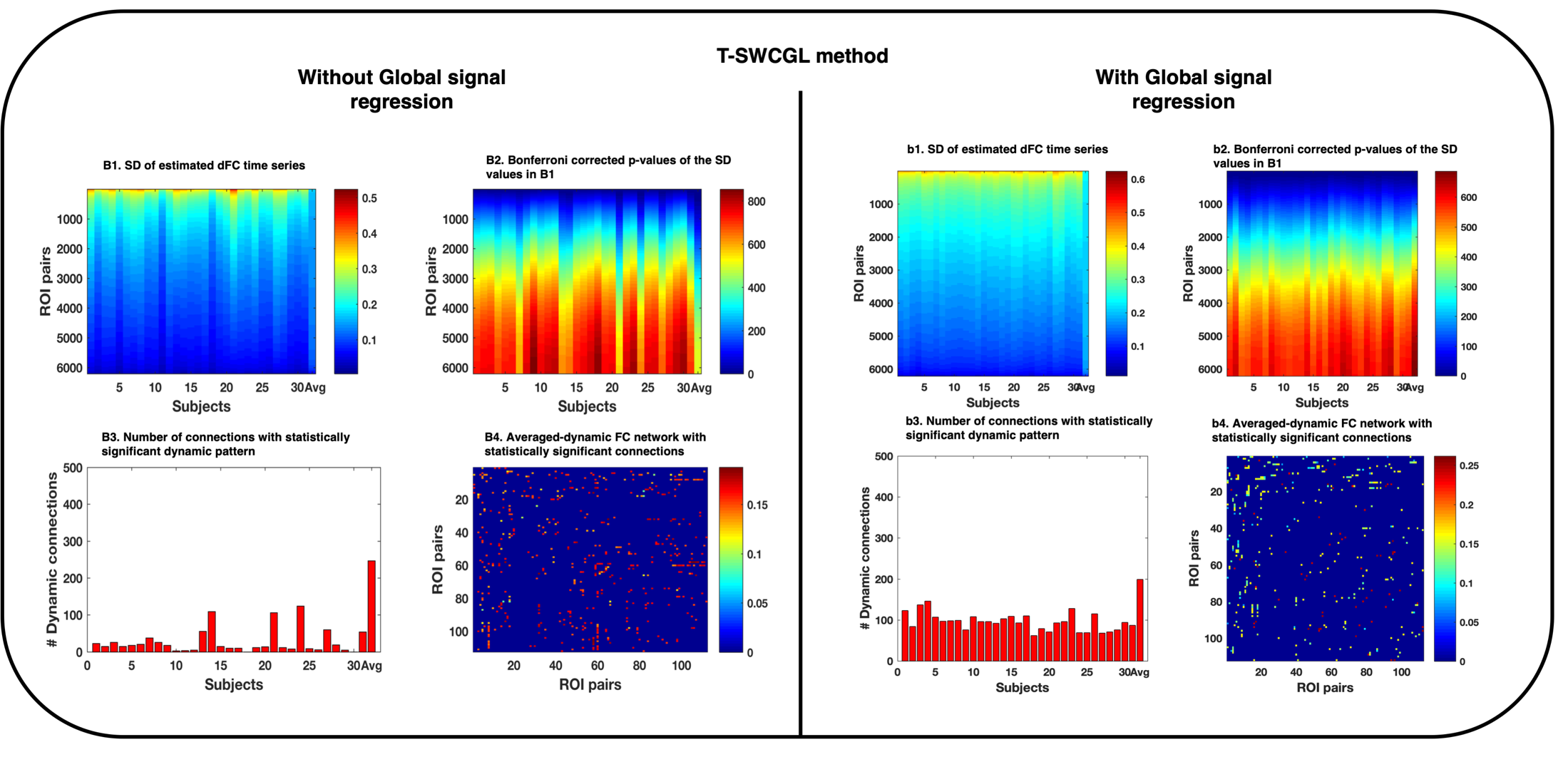


SI-Figure 2- The influence of global signal regression on the results of T-SWCGL with window length of 100 sec. The results showed that GSR considerably increased the dynamic detection power of single subjects, while dynamic detection power also was increased by subject-averaging of the measures.


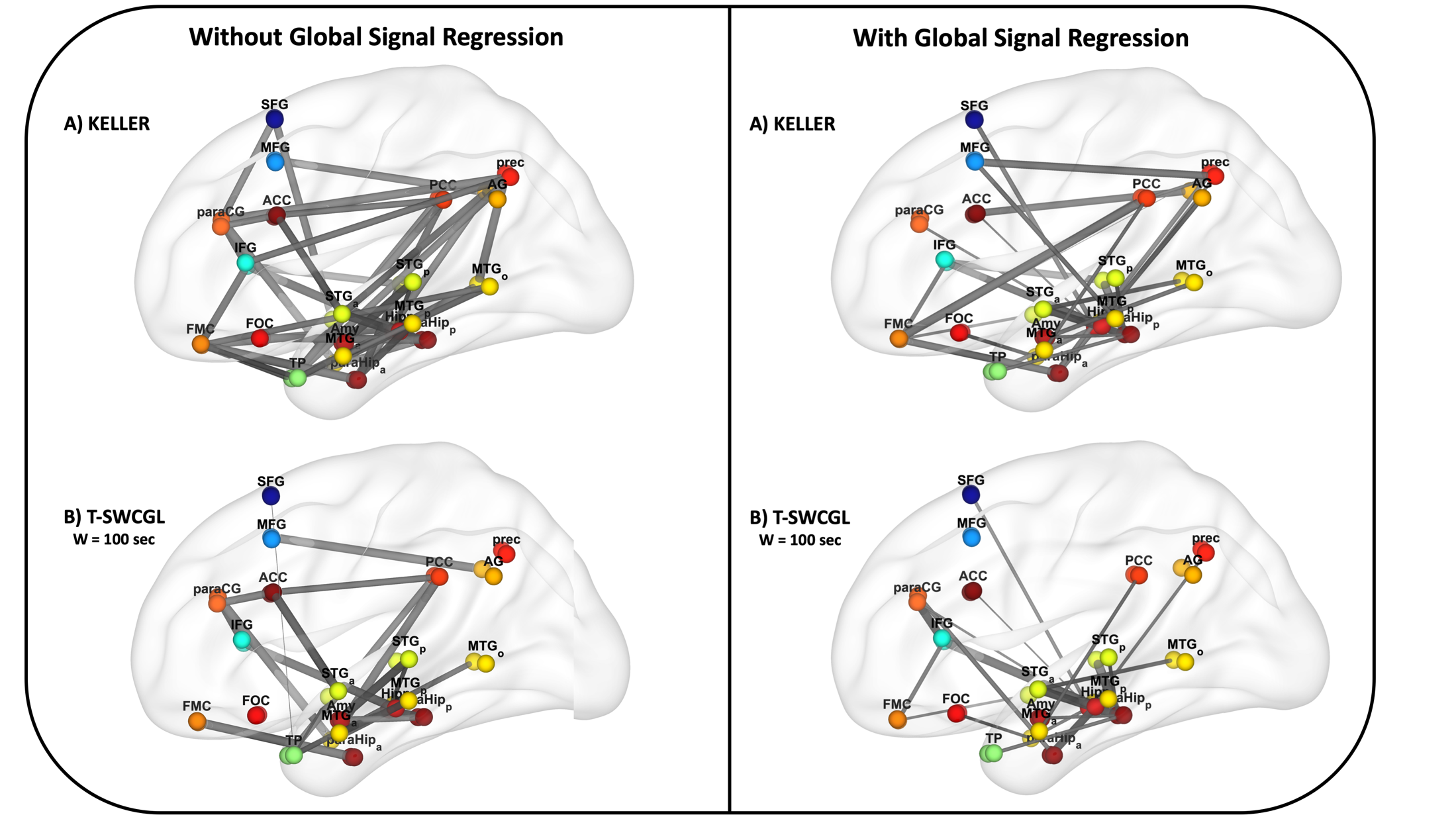


SI-Figure 3- The influence of global signal regression on the mean dynamic pattern of default mode network for the averaged case estimated by KELLER and T-SWCGL with window length of 100 sec. The results showed that there is a notable change of the results with considering global signal regression. In fact, the number of dynamic connections considerably decreased in both methods with considering global signal regression, because global signal increases the dependencies between brain regions.
